# Joint trajectories of frailty and depressive symptoms and risk of incident osteoarthritis in middle-aged and older adults

**DOI:** 10.3389/fpubh.2026.1873124

**Published:** 2026-08-06

**Authors:** Tingjie Chang, Zhipeng Qin, Feng Zhao, Ke Han, Yaohui Cui, Lincheng Duan, Jinying Hao

**Affiliations:** 1 Heping Hospital Affiliated to Changzhi Medical College, Changzhi, China; 2 Chengdu University of Traditional Chinese Medicine, Chengdu, China

**Keywords:** aging, depressive symptoms, frailty, incident osteoarthritis, trajectory

## Abstract

**Background:**

Frailty frequently overlaps with depressive symptoms among middle-aged and older adults. However, it remains unclear how these two conditions develop concurrently over time and whether their combined longitudinal patterns are associated with the subsequent onset of osteoarthritis (OA). This study aimed to characterize the joint trajectories of frailty and depressive symptoms and to evaluate their prospective association with incident OA.

**Methods:**

We analyzed 3,822 participants from the Health and Retirement Study (HRS) and 3,913 from the English Longitudinal Study of Ageing (ELSA). All participants were free of self-reported osteoarthritis (OA) at the analytical baseline. Frailty and depressive symptoms were assessed at four biennial waves using the frailty index (FI) and the 8-item Center for Epidemiologic Studies Depression Scale (CES-D-8), respectively. Group-based multi-trajectory modeling identified joint longitudinal patterns within each cohort. Cox proportional hazards models estimated hazard ratios (HRs) and 95% confidence intervals (CIs) for incident OA during a median follow-up of approximately 8 years.

**Results:**

Three similar joint trajectory groups were identified in both cohorts: persistently low, moderate, and high combined frailty and depressive symptom burden. In fully adjusted HRS models, the HRs were 1.50 (95% CI, 1.28–1.76) for the moderate-burden group and 2.19 (95% CI, 1.68–2.86) for the high-burden group. The corresponding HRs in ELSA were 1.87 (95% CI, 1.60–2.17) and 3.40 (95% CI, 2.74–4.21). Sensitivity analyses using logistic regression, first-wave event exclusion, complete cases, and a four-group trajectory model produced broadly consistent associations.

**Conclusion:**

Persistently higher combined frailty and depressive symptom burden was prospectively associated with incident OA in two ageing cohorts. These findings describe an epidemiological association and do not establish causal, preventive, predictive, or treatment effects.

## 1 Introduction

Osteoarthritis (OA) represents a major musculoskeletal disorder in ageing societies and is a leading source of chronic pain, functional decline, and disability among adults in middle and later life (1, 2). According to estimates from the Global Burden of Disease study, nearly 595 million people were living with OA worldwide in 2020, and the number of affected individuals is projected to rise steadily through 2050 (3). Beyond traditional risk factors, including older age, female sex, genetic susceptibility, excess body weight, and previous joint injury (4), OA is increasingly recognized as a condition influenced by systemic ageing processes. Age-related biological vulnerability, such as persistent inflammation and metabolic dysregulation, may accelerate OA onset and contribute to disease progression (1).

Frailty and depressive symptoms are important geriatric-related problems that commonly affect individuals in middle and older adulthood (5, 6). Frailty reflects a state of age-related biological vulnerability, characterized by depleted physiological reserve, dysfunction across multiple systems, and reduced resilience when facing external or internal stressors (5, 7). This condition has been linked to a broad spectrum of poor outcomes, including increased risks of falls, disability, hospitalization, and mortality (8, 9). Similarly, depressive symptoms are widespread in later life and are closely connected with cognitive decline, coexisting chronic diseases, and deterioration in functional capacity (6, 10).

Frailty and depressive symptoms are interconnected age-related conditions that often coexist and may reinforce each other over time (11). Their coexistence has been associated with several adverse ageing-related outcomes (12–14). Both conditions may also be associated with OA through shared biological and behavioral pathways (1). Chronic systemic inflammation is one plausible link between physical vulnerability, psychological distress, and joint degeneration (15). Sarcopenia, reduced muscle strength, sedentary behavior, and other health-related behaviors may also contribute to OA susceptibility (16–18). Recent prospective studies have reported that greater baseline frailty is associated with a higher incidence of OA (19, 20).

However, most existing studies have treated frailty or depressive symptoms as static baseline exposures, which may not adequately capture their dynamic changes over the course of aging. Moreover, because frailty and depressive symptoms frequently coexist and interact, examining either factor in isolation may underestimate the impact of their joint evolution on OA risk. Both frailty and depressive symptoms have been shown to exhibit substantial longitudinal heterogeneity (21, 22). Trajectory-based approaches can identify subgroups with similar developmental patterns over time and may therefore provide a more informative framework for understanding long-term exposure profiles and their relationships with subsequent health outcomes (23).

We therefore used HRS and ELSA data to identify joint longitudinal trajectories of frailty and depressive symptoms and examine their prospective associations with incident OA (24, 25). We hypothesized that participants with persistently higher combined burden would have a higher incidence of OA than participants with consistently low levels of both measures. The study was designed to evaluate prospective associations rather than causal effects or clinical prediction performance.

## 2 Methods

### 2.1 Study design and population

Data were obtained from two publicly accessible longitudinal cohorts designed to investigate ageing-related health: the U.S. HRS and the ELSA. The HRS, initiated in 1992, enrolls a nationally representative sample of middle-aged and older adults in the United States and conducts follow-up interviews biennially (25). ELSA started in 2002 and similarly follows a nationally representative sample of adults aged 50 years or older in England through repeated two-year assessments (24). Across both cohorts, information is collected on sociodemographic background, health-related behaviors, physical functioning, overall health status, and chronic disease conditions.

**Figure 1:**
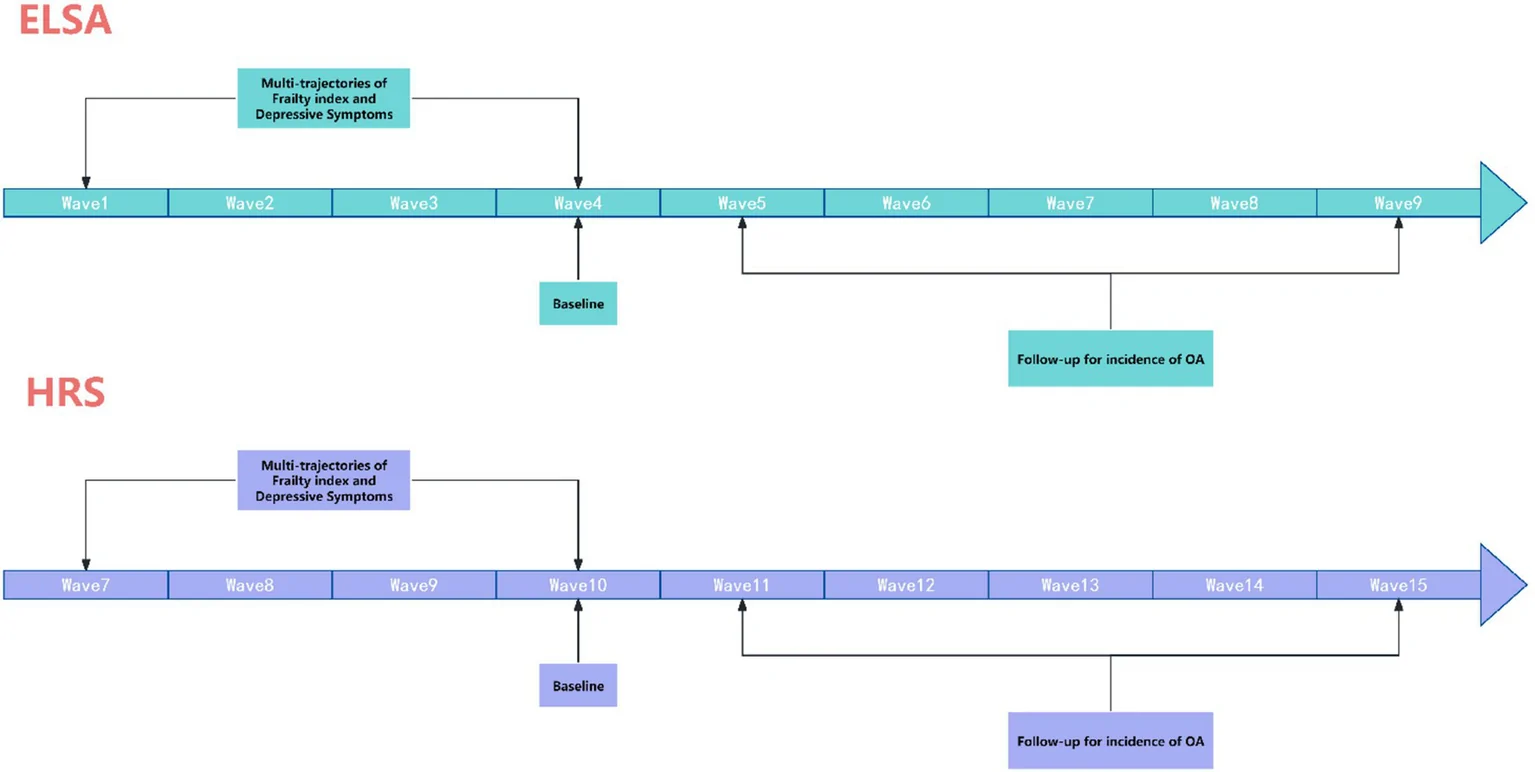
Temporal framework for defining joint frailty–depressive symptom trajectories and subsequent follow-up for incident osteoarthritis in ELSA and HRS.

Within each cohort, we first characterized the joint development of frailty and depressive symptoms and then assessed incident OA. In ELSA, trajectories were constructed from waves 1–4 (2002–2003 through 2008–2009). Wave 4 was the analytical baseline, and OA was assessed at waves 5–9 (2010–2011 through 2018–2019). In HRS, trajectories were constructed from waves 7–10 (2004 through 2010). Wave 10 was the analytical baseline, and OA was assessed at waves 11–15 (2012 through 2020) (Figure 1).

**Figure 2:**
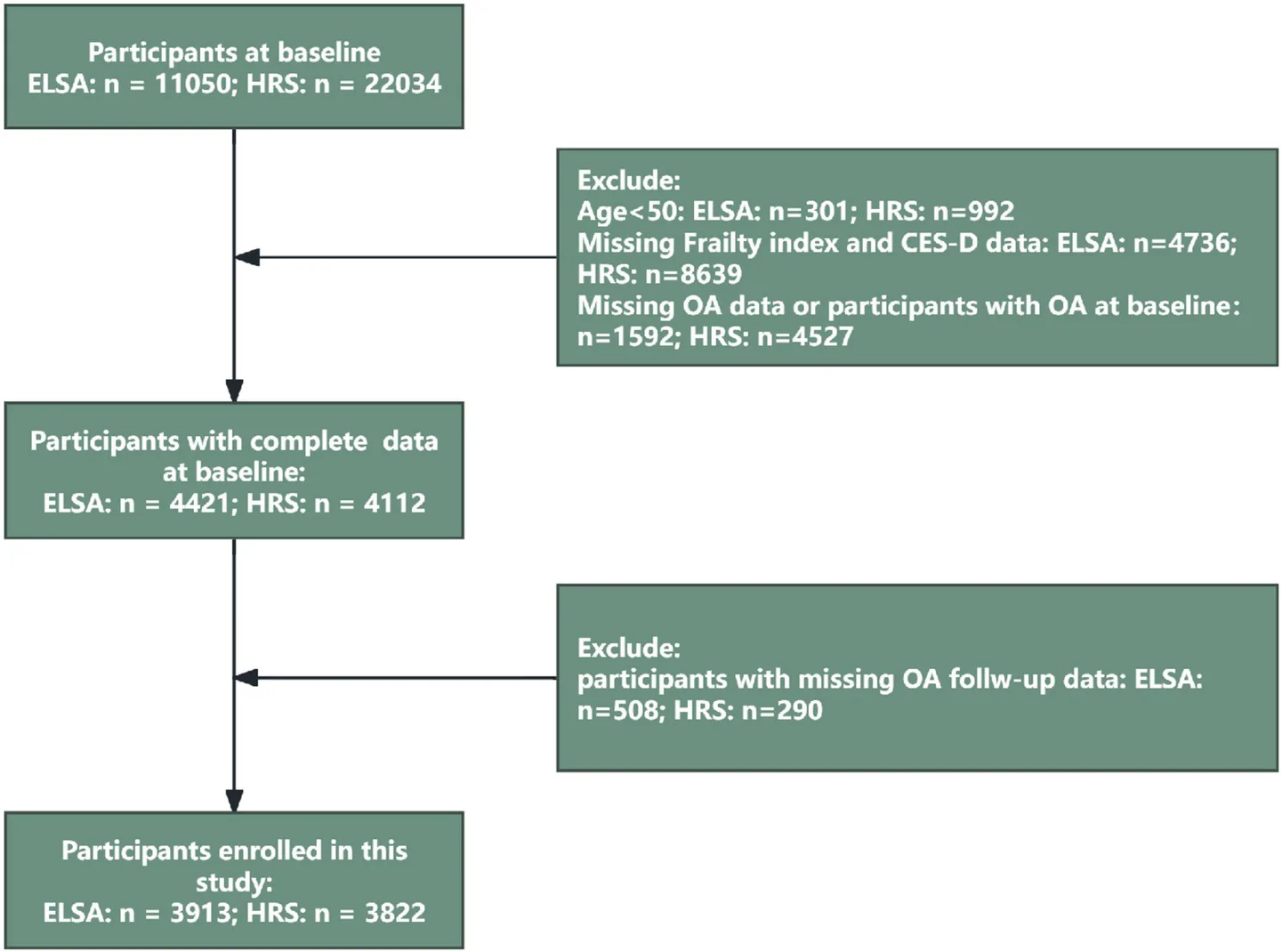
Flowchart of participant selection in the ELSA and HRS cohorts.

Eligible participants were aged 50 years or older, had no OA at the analytical baseline, provided four repeated FI and CES-D-8 measurements during the trajectory window, and completed at least one subsequent OA assessment. Participants with prevalent OA or missing exposure, outcome, or repeated trajectory data were excluded. The final samples included 3,822 HRS participants and 3,913 ELSA participants (Figure 2). Requiring four repeated measurements supported trajectory estimation but may have selected healthier and more adherent participants with more complete follow-up.

### 2.2 Exposures

Frailty was measured using an FI based on the deficit-accumulation approach (26, 27). The FI was calculated as the proportion of available health deficits present. HRS used 28 deficits and ELSA used 30 deficits, with scores ranging from 0 to 1. Higher scores indicated greater frailty. Both indices included one item on physician-diagnosed emotional, nervous, or psychiatric problems and several mobility, activities-of-daily-living, and instrumental activities-of-daily-living items. These items may overlap conceptually with depressive symptoms or preclinical OA-related functional limitation. Complete item lists and coding rules are provided in Supplementary Tables S1, S2.

Depressive symptoms were measured in both cohorts using the CES-D-8 (28). The eight binary items were summed to produce a score from 0 to 8, with higher scores indicating more depressive symptoms. The same instrument and score range were used in both cohorts, although cohort-specific source variables were harmonized for analysis.

### 2.3 Incident OA

Osteoarthritis was ascertained at each wave using a two-step algorithm. Participants were first asked whether a doctor had ever told them that they had arthritis or rheumatism. Those responding affirmatively were subsequently asked whether their arthritis was osteoarthritis. OA was defined only when both responses were affirmative. Participants who explicitly reported no arthritis, or who reported arthritis but explicitly denied having the osteoarthritis subtype, were classified as not having OA. Responses coded as “do not know,” “refused,” missing, or not ascertained for either component were treated as missing and excluded from the corresponding analysis. Among participants free of OA at baseline, incident OA was defined at the first follow-up interview at which both criteria were met. Event time was assigned using the month and year of that interview and therefore represented the first observed OA report rather than the exact date of clinical onset. Follow-up ended at the first OA report, death, loss to follow-up, or the final available assessment.

### 2.4 Covariates

Baseline covariates were selected from prior literature and included age, sex, educational level, marital status, race or ethnicity, smoking, alcohol consumption, physical activity, and body mass index (BMI). Age and BMI were modeled continuously. Categorical coding is reported in Table 1, and covariate missingness is summarized in Supplementary Table S3. BMI and physical activity were included in the fully adjusted model, but both variables may be confounders or may partly lie on pathways linking frailty and depressive symptoms to OA.

**Table 1:** Baseline characteristics by joint trajectory group in the ELSA and HRS cohorts.

Variable	Levels	Overall		Group 1		Group 2		Group 3	
		ELSA	HRS	ELSA	HRS	ELSA	HRS	ELSA	HRS
		*N* = 3,913	*N* = 3,822	*N* = 2,840	*N* = 2,631	*N* = 804	*N* = 946	*N* = 269	*N* = 245
Age, M (Q₁, Q₃)		66 (60, 74)	66 (60, 74)	64 (60,72)	65 (59,72)	71 (63,79)	70 (62,78)	71 (64,80)	69 (60,77)
BMI, M (Q₁, Q₃)		27.40 (24.80, 30.50)	26.60 (23.70, 30.10)	27.00 (24.60,30.00)	26.40 (23.60,29.45)	28.50 (25.50,31.63)	27.40 (24.22,31.50)	29.30 (25.80,33.20)	27.40 (23.60,32.20)
Duration of Follow-up, M (Q₁, Q₃)		98 (48, 120)	96 (50, 118)	116 (70, 121)	111 (67,118)	70 (28,118)	74 (42,115)	47 (25,76)	56 (25,92)
Gender, *n* (p%)									
	Female	2095 (53.54)	2003 (52.41)	1,445 (50.88)	1,390 (52.83)	481 (59.83)	472 (49.89)	169 (62.83)	141 (57.55)
	Male	1818 (46.46)	1819 (47.59)	1,395 (49.12)	1,241 (47.17)	323 (40.17)	474 (50.11)	100 (37.17)	104 (42.45)
Race, *n* (p%)									
	Others	79 (2.02)	718 (18.79)	45 (1.58)	460 (17.48)	21 (2.61)	187 (19.77)	13 (4.83)	71 (28.98)
	White	3,834 (97.98)	3,104 (81.21)	2,795 (98.42)	2,171 (82.52)	783 (97.39)	759 (80.23)	256 (95.17)	174 (71.02)
Marital, *n* (p%)									
	Married	2,622 (67.01)	2,445 (63.97)	2064 (72.68)	1780 (67.65)	437 (54.35)	546 (57.72)	121 (44.98)	119 (48.57)
	Other	1,291 (32.99)	1,377 (36.03)	776 (27.32)	851 (32.35)	367 (45.65)	400 (42.28)	148 (55.02)	126 (51.43)
Education, *n* (p%)									
	Below high school	1,509 (38.56)	538 (14.08)	904 (31.83)	249 (9.46)	431 (53.61)	205 (21.67)	174 (64.68)	84 (34.29)
	College or above	1,542 (39.41)	2060 (53.90)	1,265 (44.54)	1,593 (60.55)	221 (27.49)	386 (40.80)	56 (20.82)	81 (33.06)
	High school	862 (22.03)	1,224 (32.03)	671 (23.63)	789 (29.99)	152 (18.91)	355 (37.53)	39 (14.50)	80 (32.65)
Smoke, *n* (p%)									
	No	3,433 (87.73)	1804 (47.20)	2,543 (89.54)	1,303 (49.52)	678 (84.33)	404 (42.71)	212 (78.81)	97 (39.59)
	Yes	480 (12.27)	2018 (52.80)	297 (10.46)	1,328 (50.48)	126 (15.67)	542 (57.29)	57 (21.19)	148 (60.41)
Drink, *n* (p%)									
	<1/week	1,493 (38.15)	2,187 (57.22)	900 (31.69)	1,384 (52.60)	415 (51.62)	617 (65.22)	178 (66.17)	186 (75.92)
	≥1/week	2,420 (61.85)	1,635 (42.78)	1940 (68.31)	1,247 (47.40)	389 (48.38)	329 (34.78)	91 (33.83)	59 (24.08)
Physical activity, *n* (p%)									
	High	3,132 (80.04)	1,474 (38.57)	2,533 (89.19)	1,197 (45.50)	516 (64.18)	247 (26.11)	83 (30.86)	30 (12.24)
	Low	781 (19.96)	2,348 (61.43)	307 (10.81)	1,434 (54.50)	288 (35.82)	699 (73.89)	186 (69.14)	215 (87.76)
Incidence of OA, *n* (p%)									
	No	2,889 (73.83)	2,935 (76.79)	2,245 (79.05)	2051 (77.96)	514 (63.93)	707 (74.74)	130 (48.33)	177 (72.24)
	Yes	1,024 (26.17)	887 (23.21)	595 (20.95)	580 (22.04)	290 (36.07)	239 (25.26)	139 (51.67)	68 (27.76)

### 2.5 Statistical analysis

Baseline characteristics were summarized separately by cohort and trajectory group. Normally distributed continuous variables are reported as means and standard deviations, skewed continuous variables as medians and interquartile ranges, and categorical variables as counts and percentages.

Joint FI and CES-D-8 trajectories were identified using group-based multi-trajectory modeling (GBMTM). Two-, three-, and four-group models with linear, quadratic, or cubic polynomial terms were evaluated. Model assessment considered the Bayesian information criterion (BIC), Akaike information criterion, entropy, average posterior probability (AvePP), odds of correct classification, class size, interpretability, parsimony, and cross-cohort comparability. Four-group models improved BIC, and one HRS four-group specification retained all groups above 5%. However, the ELSA four-group solution included a group below 5%. The three-group linear solution produced similar and clinically interpretable patterns in both cohorts, with entropy values of 0.925 in HRS and 0.936 in ELSA. Group-specific AvePP values ranged from 93.7 to 97.6% in HRS and from 94.0 to 98.0% in ELSA. We therefore selected the three-group linear solution for the primary cross-cohort analysis and evaluated the four-group solution in sensitivity analyses. Participants were assigned to the group with the highest posterior probability.

Cox proportional hazards models estimated associations between trajectory group and incident OA. The low-burden trajectory was the reference. Model 1 was unadjusted, Model 2 adjusted for age and sex, and Model 3 additionally adjusted for education, marital status, smoking, physical activity, race or ethnicity, alcohol consumption, and BMI. Death and loss to follow-up were treated as censoring events. The proportional hazards assumption was evaluated using Schoenfeld residuals.

Subgroup analyses and interaction terms were used to evaluate potential effect modification. Missing covariate data were imputed separately within HRS and ELSA using multiple imputation by chained equations, with random forest as the conditional imputation method, implemented in the mice package in R. Five imputed datasets were generated, and estimates were pooled according to Rubin’s rules. FI and CES-D-8 trajectory measurements were not imputed; participants without all four repeated measurements were excluded. GBMTM analyses were conducted in SAS version 9.4, and all other analyses were conducted in R version 4.2.1. All tests were two-sided, with *p* < 0.05 considered statistically significant.

## 3 Results

### 3.1 Baseline characteristics

3,822 participants from HRS and 3,913 participants from ELSA were included in the final analysis. In the HRS cohort, the median age of participants was 66 years, and 52.41% were women. In the ELSA cohort, the median age was also 66 years, and 53.54% were women. Detailed baseline characteristics of both cohorts are displayed in Table 1.

### 3.2 Joint trajectories of frailty and depressive symptoms

**Figure 3:**
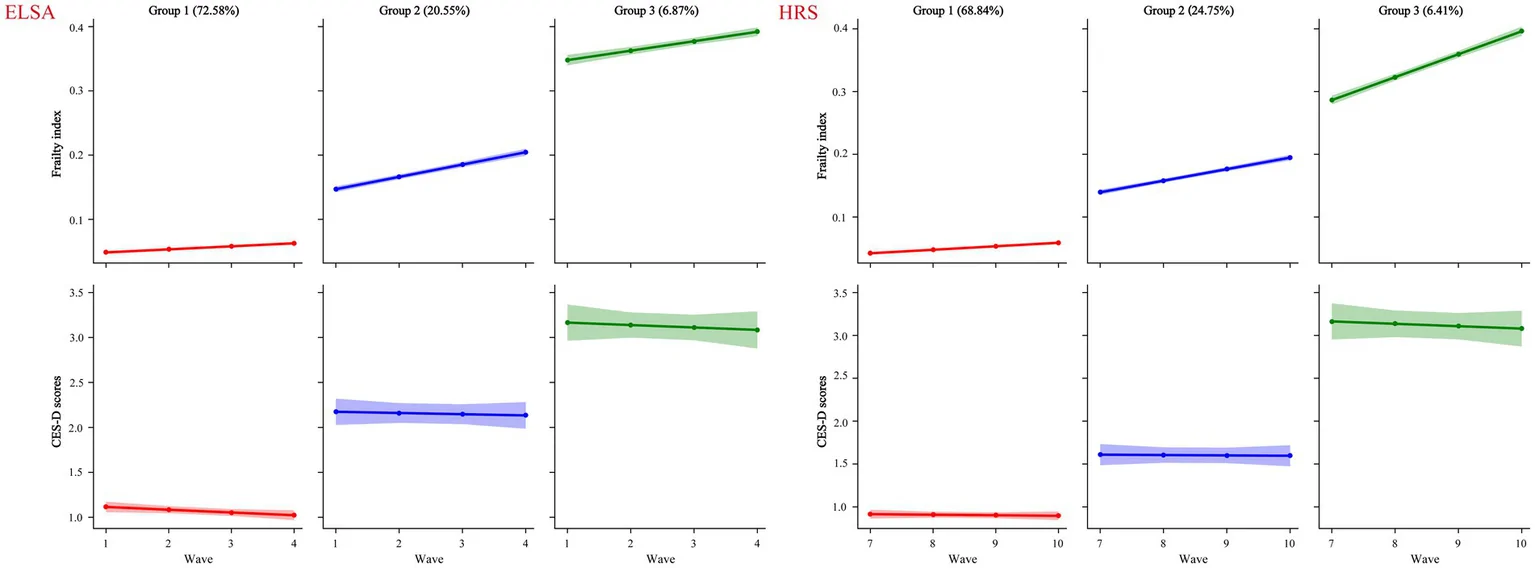
Joint trajectories of frailty index and depressive symptoms in the ELSA and HRS cohorts.

Using GBMTM, we identified three similar joint trajectories of frailty and depressive symptoms in both HRS and ELSA (Figure 3). Trajectory 1, labeled persistent low frailty-low depression, was the largest group in both cohorts, comprising 68.84% of participants in HRS and 72.58% in ELSA. Participants in this group maintained consistently low levels of both frailty index and depressive symptom scores throughout the trajectory-building period.

Trajectory 2, labeled persistent moderately high frailty-moderate depression, accounted for 24.75% of participants in HRS and 20.55% in ELSA. Individuals in this group had persistently higher frailty and depressive symptom levels than those in Trajectory 1, with a modest upward trend over time.

Trajectory 3, labeled persistent high frailty-high depression, was the smallest group in both cohorts, representing 6.41% of participants in HRS and 6.87% in ELSA. Participants in this group showed high levels of frailty and depressive symptoms from the outset, which remained elevated and tended to worsen over time. Further details of the trajectory model parameters are presented in Supplementary Tables S4–S11.

### 3.3 Associations between joint trajectories and incident OA

During a median follow-up of 8.0 years, 887 HRS participants and 1,024 ELSA participants reported incident OA. In HRS, events occurred in 580 of 2,631 low-burden participants (22.04%), 239 of 946 moderate-burden participants (25.26%), and 68 of 245 high-burden participants (27.76%). Relative to the low-burden group, the crude risk differences were 3.22 and 5.72 percentage points, respectively. In ELSA, events occurred in 595 of 2,840 low-burden participants (20.95%), 290 of 804 moderate-burden participants (36.07%), and 139 of 269 high-burden participants (51.67%). The corresponding crude risk differences were 15.12 and 30.72 percentage points. Cox regression results are presented in Table 2.

**Table 2:** Hazard ratios for incident OA according to joint trajectory group in the ELSA and HRS cohorts.

Variables	Model 1		Model 2		Model 3		Model 4	
	HR (95% CI)	*p*	HR (95% CI)	*p*	HR (95% CI)	*p*	HR (95% CI)	*p*
ELSA								
Group								
1	1.00 (Reference)		1.00 (Reference)		1.00 (Reference)		1.00 (Reference)	
2	2.26 (1.96–2.60)	<0.001	2.00 (1.73–2.32)	<0.001	1.95 (1.64–2.31)	<0.001	1.87 (1.60–2.17)	<0.001
3	4.30 (3.57–5.17)	<0.001	3.74 (3.09–4.53)	<0.001	3.79 (3.00–4.78)	<0.001	3.40 (2.74–4.21)	<0.001
HRS								
Group								
1	1.00 (Reference)		1.00 (Reference)		1.00 (Reference)		1.00 (Reference)	
2	1.48 (1.27–1.72)	<0.001	1.45 (1.25–1.69)	<0.001	1.58 (1.35–1.85)	<0.001	1.50 (1.28–1.76)	<0.001
3	2.04 (1.59–2.63)	<0.001	2.01 (1.56–2.58)	<0.001	2.36 (1.81–3.08)	<0.001	2.19 (1.68–2.86)	<0.001

HR, hazard ratio; CI, confidence interval.

Model 1: Crude.

Model 2: Adjusted for age and gender.

Model 3: Adjusted for age, gender, race, marital status, education, smoking, and alcohol consumption.

Model 4: Model 3 plus BMI and physical activity.

Model 3 adjusted for age, gender, race, marital status, education, smoking, and alcohol consumption, but excluded BMI and physical activity. In HRS, the HRs were 1.58 (95% CI, 1.35–1.85) for the moderate-burden group and 2.36 (95% CI, 1.81–3.08) for the high-burden group. In ELSA, the corresponding HRs were 1.95 (95% CI, 1.64–2.31) and 3.79 (95% CI, 3.00–4.78).

**Figure 4:**
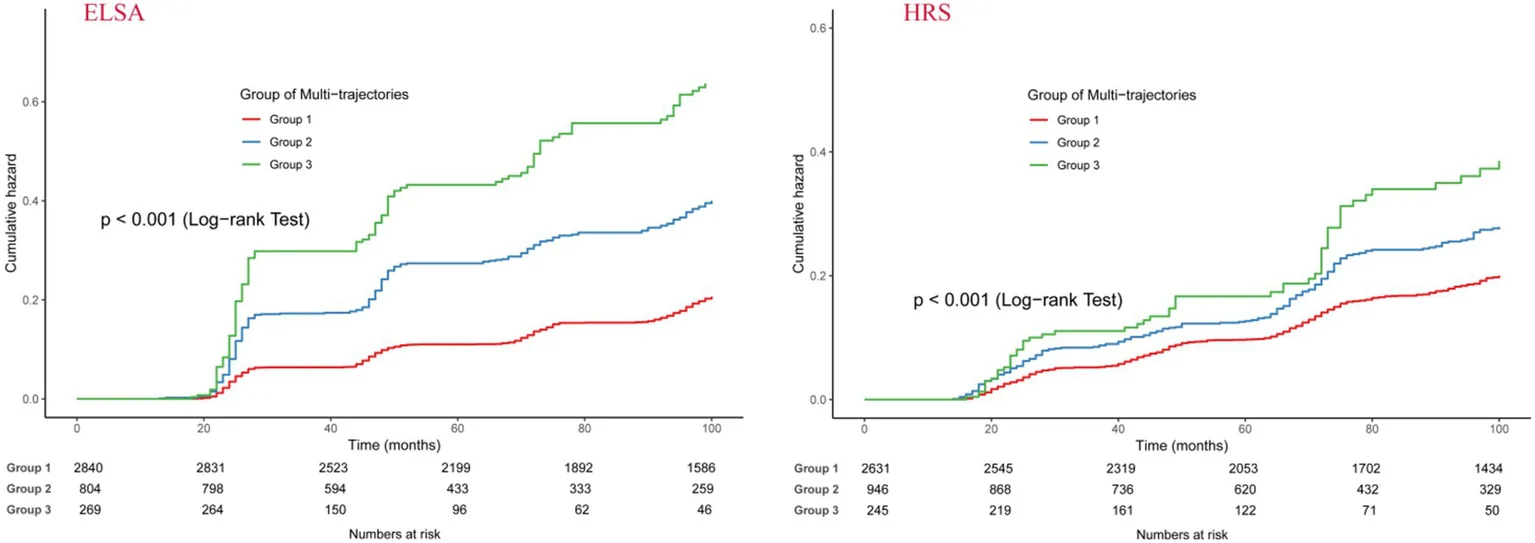
Cumulative hazard of incident osteoarthritis according to joint trajectory group in the ELSA and HRS cohorts.

Further adjustment for baseline BMI and physical activity in Model 4 modestly attenuated these estimates. In HRS, the HRs were 1.50 (95% CI, 1.28–1.76) and 2.19 (95% CI, 1.68–2.86), respectively. In ELSA, the corresponding HRs were 1.87 (95% CI, 1.60–2.17) and 3.40 (95% CI, 2.74–4.21). All four adjusted associations remained statistically significant (*p* < 0.001). Point estimates were larger in ELSA than in HRS at both burden levels. Within each cohort, point estimates were larger at higher combined burden levels, although no formal trend test was performed. Cumulative hazard curves are shown in Figure 4, and the proportional hazards assumption was satisfied. The global Schoenfeld residual test was nonsignificant (*p* > 0.05).

### 3.4 Subgroup analyses

**Figure 5:**
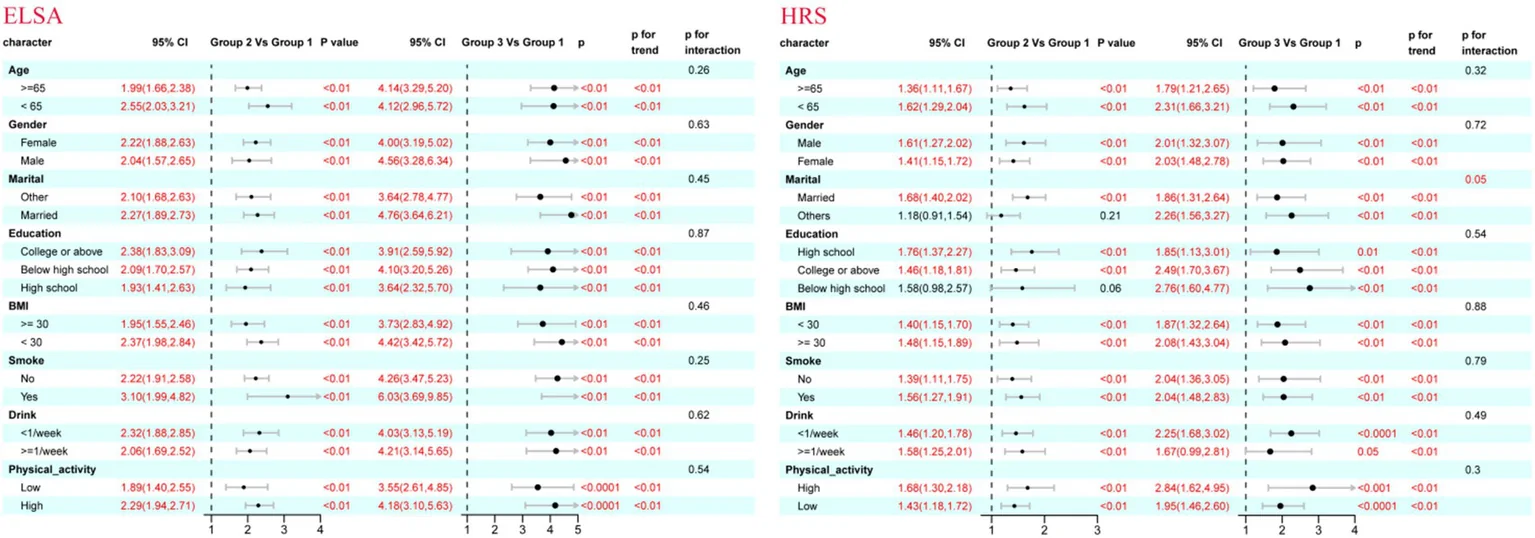
Subgroup analyses of the association between joint trajectory group and incident OA in ELSA and HRS.

Across most subgroups, the higher-burden trajectories remained associated with incident OA in both cohorts (Figure 5). In HRS, the interaction *p* value for marital status was 0.05, whereas other interaction tests were not statistically significant. Because several subgroup comparisons were performed, the marital-status result was considered exploratory and was not interpreted as strong evidence of effect modification. No statistically significant interactions were observed in ELSA.

### 3.5 Sensitivity analyses

Four sensitivity analyses were conducted. Logistic regression produced similar directions of association (Supplementary Table S12). Excluding participants who reported OA at the first post-baseline wave also produced similar associations (Supplementary Table S13). Complete-case analyses were consistent with the primary models (Supplementary Table S14). A four-group GBMTM specification produced broadly consistent associations (Supplementary Table S15 and Supplementary Figure 1). However, HRS estimates were not monotonic across the two intermediate four-group categories. The four-group analysis therefore does not support a simple dose–response interpretation.

## 4 Discussion

In HRS and ELSA, three similar joint trajectories of frailty and depressive symptoms were prospectively associated with incident OA. In the primary three-group models, point estimates were larger for the high-burden group than for the moderate-burden group. The direction of association was consistent across cohorts and sensitivity analyses. However, the observational design, absence of a formal trend test, and non-monotonic HRS four-group results preclude causal or simple dose–response interpretations.

These findings extend previous work by jointly characterizing repeated frailty and depressive symptom measures. Earlier studies reported bidirectional relationships between frailty and depressive symptoms (12, 13) and associated their coexistence with adverse outcomes (11, 29–31). This combined vulnerability has been described as a depressed-frail phenotype (11, 32, 33). Our results show that persistent combined burden was associated with later OA reporting in two cohorts. Joint trajectories may therefore complement baseline measures when describing longitudinal heterogeneity, although their incremental predictive value was not evaluated.

Several biological and behavioral pathways may help explain the association between joint frailty–depressive symptom trajectories and incident OA. Chronic systemic low-grade inflammation may represent a key common pathway linking frailty, depressive symptoms, and OA. Previous research has reported that frailty and depressive symptoms are each accompanied by elevated inflammatory biomarkers, including IL-6, CRP, and TNF-α (15, 34). This age-related state of persistent low-grade inflammation has been described as “inflammaging” (35, 36). Such a pro-inflammatory milieu may disrupt cartilage homeostasis, promote cartilage degradation, and drive synovial inflammation, thereby contributing to the development and progression of OA (1, 37). Accordingly, individuals in unfavorable frailty-depressive symptom trajectories may be more susceptible to joint tissue damage when exposed to persistent inflammatory burden over time.

Behavioral and biomechanical pathways may further amplify this risk. Frailty is often accompanied by sarcopenia and reduced muscle strength, which can compromise joint stability and alter mechanical load distribution (38). Depressive symptoms, in turn, are frequently associated with reduced activity and increased fatigue, which may further accelerate muscle decline and weight gain (14). Given that obesity is a major modifiable risk factor for OA, frailty and depressive symptoms may jointly contribute to OA through interconnected processes involving muscle deterioration, reduced physical activity, and increased metabolic burden (1).

Several limitations should be considered. OA was identified from self-reported physician diagnoses; joint site and severity were unavailable, and interview dates only approximated onset. Requiring four repeated exposure assessments may also have preferentially retained healthier, more adherent participants, and no included-versus-excluded comparison or attrition weighting was undertaken. These features may introduce outcome misclassification and selection bias and limit generalizability.

The FI included psychiatric and functional deficits that may overlap with CES-D-8 symptoms or preclinical OA-related limitations, so residual confounding and reverse causation cannot be excluded. BMI and physical activity may also lie partly on the causal pathway, making overadjustment possible. In addition, trajectory-group assignment was treated as fixed without propagating classification uncertainty, death was censored without competing-risk analysis, and interval-censored OA onset was not modeled. Differences in cohort composition, follow-up, outcome reporting, and calendar period may partly explain the larger ELSA estimates and limit direct cross-cohort comparison.

The joint trajectories describe longitudinal heterogeneity in frailty and depressive symptom burden, but their incremental predictive performance was not evaluated. The findings therefore support a prospective epidemiological association and do not establish clinical risk-stratification, prevention, or treatment benefits. Independent validation and purpose-designed prediction or intervention studies are required before clinical application.

## 5 Conclusion

Frailty and depressive symptoms followed heterogeneous joint trajectories in middle-aged and older adults. Persistently higher combined burden was prospectively associated with incident OA in HRS and ELSA. These observational findings do not establish causality or preventive benefit, and their predictive and clinical utility requires dedicated validation.

## Data Availability

The original contributions presented in the study are included in the article/Supplementary material, further inquiries can be directed to the corresponding author.
