# Supplementary material for: Joint trajectories of frailty and depressive symptoms and risk of incident osteoarthritis in middle-aged and older adults
Source: Front Public Health. 2026 Aug 6;14:1873124. doi: 10.3389/fpubh.2026.1873124 (PMC13489894; doi:10.3389/fpubh.2026.1873124)
Supplement: Supplementary file 1 [file Supplementary_file_1.docx]

**Joint trajectories of frailty and depressive symptoms and risk of incident osteoarthritis in middle-aged and older adults**

**Supplemental Tables S1.** Variables used to construct the frailty index in ELSA.

**Supplemental Tables S2.** Variables used to construct the frailty index in HRS.

**Supplemental Tables S3.** The numbers (percentages) of participants with missing covariates.

**Supplemental Tables S4.** Evaluation Indicators of the fitting effect of multiple models in ELSA.

**Supplemental Tables S5.** Multiple model trajectory group evaluation indicators in ELSA.

**Supplemental Tables S6.** Trajectory parameter estimation for the model in ELSA.

**Supplemental Tables S7**. The best trajectory model applicability in ELSA.

**Supplemental Tables S8**. Evaluation Indicators of the fitting effect of multiple models in HRS.

**Supplemental Tables S9**. Multiple model trajectory group evaluation indicators in HRS.

**Supplemental Tables S10**. Trajectory parameter estimation for the model in HRS.

**Supplemental Tables S11**. Best trajectory model applicability in HRS.

**Supplemental Tables S12**. Sensitivity analysis of the association between joint trajectories of frailty index and depressive symptoms and incident OA using multivariable logistic regression.

**Supplemental Tables S13.** Sensitivity analysis of the association between joint trajectories of frailty index and depressive symptoms and incident OA after excluding participants who developed OA during the first follow-up wave.

**Supplemental Tables S14.** Sensitivity analysis of the association between joint trajectories of frailty index and depressive symptoms and incident OA using complete-case analysis without imputation of missing covariate data.

**Supplemental Tables S15.** Sensitivity analysis of the association between joint trajectories of frailty index and depressive symptoms and incident OA using a four-group trajectory model.

**Supplemental Figure 1.** Sensitivity analysis using a four-group multi-trajectory model of frailty index and depressive symptoms
**Supplemental Tables S1.** Variables used to construct the frailty index in ELSA.

| **No** | **Description of the items** | **Cut-off value** |
| --- | --- | --- |
| 1 | Self-reported diagnosis of hypertension by a doctor | Yes = 1, No = 0 |
| 2 | Self-reported diagnosis of diabetes by a doctor |  |
| 3 | Self-reported diagnosis of heart attack, coronary heart disease, angina, congestive heart failure, or other heart problems by a doctor |  |
| 4 | Self-reported diagnosis of stroke by a doctor |  |
| 5 | Self-reported diagnosis of cancer by a doctor |  |
| 6 | Self-reported diagnosis of chronic lung diseases by a doctor |  |
| 7 | Self-reported diagnosis of asthma by a doctor |  |
| 8 | Self-reported diagnosis of emotional, nervous, or psychiatric problems by a doctor |  |
| 9 | Self-reported diagnosis of memory-related disease by a doctor |  |
| 10 | Self-reported vision problems |  |
| 11 | Self-reported hearing problems |  |
| 12 | Difficulty with dressing | Did not have any problems with the activity=0; some difficulty with the activity or could not do the activity=1. |
| 13 | Difficulty with bathing or showering |  |
| 14 | Difficulty with eating |  |
| 15 | Difficulty with getting in and out of bed |  |
| 16 | Difficulty with using the toilet |  |
| 17 | Difficulty with managing money |  |
| 18 | Difficulty with taking medications |  |
| 19 | Difficulty with shopping for groceries |  |
| 20 | Difficulty with preparing meals |  |
| 21 | Difficulty with doing housework |  |
| 22 | Difficulty with walking 100 yards |  |
| 23 | Difficulty with getting up from a chair after sitting for long periods |  |
| 24 | Difficulty with climbing several flights of stairs without resting |  |
| 25 | Difficulty with lifting or carrying weights over 10 pounds/ jins |  |
| 26 | Difficulty with picking up a coin from the table |  |
| 27 | Difficulty with stooping, kneeling, or crouching |  |
| 28 | Difficulty with reaching arms above shoulder level |  |
| 29 | Self reported health | Poor/ fair = 1, excellent/ very good/ or good = 0 |
| 30 | Cognition: (memory test score + orientation test score) **/** 14 | Continuous, ranging from 0 to 1 |

The memory score is calculated as the average of words that are not remembered in the immediate and delayed word recall tasks. The memory score ranges from 0 to 10. The orientation test consists of four four questions about the day of the week, the month, the date of the month, and the year. Each incorrect response earns one point, resulting in a scoring range from 0 to 4. Thus, the sum of memory and orientation test scores was divided by 14 to yield a continuous cognitive variable, ranging from 0 to 1.

**Supplemental Tables S2.** Variables used to construct the frailty index in HRS.

| **No** | **Description of the items** | **Cut-off value** |
| --- | --- | --- |
| 1 | Self-reported diagnosis of hypertension by a doctor | Yes = 1, No = 0 |
| 2 | Self-reported diagnosis of diabetes by a doctor |  |
| 3 | Self-reported diagnosis of heart attack, coronary heart disease, angina, congestive heart failure, or other heart problems by a doctor |  |
| 4 | Self-reported diagnosis of stroke by a doctor |  |
| 5 | Self-reported diagnosis of cancer by a doctor |  |
| 6 | Self-reported diagnosis of chronic lung diseases by a doctor |  |
| 7 | Self-reported diagnosis of emotional, nervous, or psychiatric problems by a doctor |  |
| 8 | Self-reported vision problems |  |
| 9 | Self-reported hearing problems |  |
| 10 | Difficulty with dressing | Did not have any problems with the activity=0; some difficulty with the activity or could not do the activity=1. |
| 11 | Difficulty with bathing or showering |  |
| 12 | Difficulty with eating |  |
| 13 | Difficulty with getting in and out of bed |  |
| 14 | Difficulty with using the toilet |  |
| 15 | Difficulty with managing money |  |
| 16 | Difficulty with taking medications |  |
| 17 | Difficulty with shopping for groceries |  |
| 18 | Difficulty with preparing meals |  |
| 29 | Difficulty with doing housework |  |
| 20 | Difficulty with walking 100 yards |  |
| 21 | Difficulty with getting up from a chair after sitting for long periods |  |
| 22 | Difficulty with climbing several flights of stairs without resting |  |
| 23 | Difficulty with lifting or carrying weights over 10 pounds/ jins |  |
| 24 | Difficulty with picking up a coin from the table |  |
| 25 | Difficulty with stooping, kneeling, or crouching |  |
| 26 | Difficulty with reaching arms above shoulder level |  |
| 27 | Self reported health | Poor/ fair = 1, excellent/ very good/ or good = 0 |
| 28 | Cognition: (memory test score + orientation test score) **/** 14 | Continuous, ranging from 0 to 1 |

The memory score is calculated as the average of words that are not remembered in the immediate and delayed word recall tasks. The memory score ranges from 0 to 10. The orientation test consists of four four questions about the day of the week, the month, the date of the month, and the year. Each incorrect response earns one point, resulting in a scoring range from 0 to 4. Thus, the sum of memory and orientation test scores was divided by 14 to yield a continuous cognitive variable, ranging from 0 to 1.

**Supplemental Tables S3.** The numbers (percentages) of participants with missing covariates.

| **Covariate** | **Number** | | **Percentage (%)** | |
| --- | --- | --- | --- | --- |
|  | ELSA | HRS | ELSA | HRS |
| BMI | 564 | 40 | 14.41 | 1.05 |
| Education | - | 1 | - | 0.03 |
| Physical activity | 1 | 5 | 0.03 | 0.13 |
| Smoke | 39 | 24 | 1 | 0.63 |
| Alcohol consumption | 421 | 8 | 10.76 | 0.21 |

**Supplemental Tables S4.** Evaluation Indicators of the fitting effect of multiple models in ELSA.

| **Model ID** | ***Log(LL)*** | ***BIC*** | ***AIC*** | ***E_k_*** |
| --- | --- | --- | --- | --- |
| M1[11][11] | -11964.491 | -12009.987 | -11975.491 | 0.969 |
| M2[22][22] | -11956.593 | -12018.634 | -11971.593 | 0.969 |
| M3[33][33] | -11945.814 | -12024.399 | -11964.814 | 0.969 |
| M4[111][111] | -9970.581 | -10036.758 | -9986.581 | 0.936 |
| M5[222][222] | -9961.321 | -10052.313 | -9983.321 | 0.936 |
| M6[333][333] | -9942.331 | -10058.140 | -9970.331 | 0.937 |
| M7[1111][1111] | -9044.443 | -9131.299 | -9065.443 | 0.920 |
| M8[2222][2222] | -9032.655 | -9152.600 | -9061.655 | 0.920 |
| M9[3333][3333] | -9010.016 | -9163.049 | -9047.016 | 0.921 |

**Supplemental Tables S5.** Multiple model trajectory group evaluation indicators in ELSA.

| **Model ID** | **Class** | ***OCC*** | ***AvepP*** | ***P*** | ***π*** |
| --- | --- | --- | --- | --- | --- |
| M1[11][11] | Class1 | 31.250 | 99.452 | 85.357 | 85.306 |
| M1[11][11] | Class2 | 198.038 | 97.152 | 14.643 | 14.694 |
| M2[22][22] | Class1 | 31.910 | 99.463 | 85.331 | 85.298 |
| M2[22][22] | Class2 | 194.006 | 97.096 | 14.669 | 14.702 |
| M3[33][33] | Class1 | 30.619 | 99.439 | 85.331 | 85.263 |
| M3[33][33] | Class2 | 200.848 | 97.200 | 14.669 | 14.737 |
| M4[111][111] | Class1 | 18.496 | 97.954 | 72.579 | 72.132 |
| M4[111][111] | Class2 | 59.448 | 94.038 | 20.547 | 20.970 |
| M4[111][111] | Class3 | 555.503 | 97.628 | 6.875 | 6.898 |
| M5[222][222] | Class1 | 18.310 | 97.929 | 72.553 | 72.086 |
| M5[222][222] | Class2 | 60.565 | 94.148 | 20.496 | 20.987 |
| M5[222][222] | Class3 | 483.417 | 97.296 | 6.951 | 6.927 |
| M6[333][333] | Class1 | 18.518 | 97.958 | 72.604 | 72.145 |
| M6[333][333] | Class2 | 61.331 | 94.207 | 20.470 | 20.958 |
| M6[333][333] | Class3 | 475.435 | 97.239 | 6.926 | 6.896 |
| M7[1111][1111] | Class1 | 16.392 | 96.765 | 64.988 | 64.600 |
| M7[1111][1111] | Class2 | 32.482 | 90.823 | 23.102 | 23.354 |
| M7[1111][1111] | Class3 | 227.145 | 95.622 | 8.638 | 8.772 |
| M7[1111][1111] | Class4 | 879.316 | 96.749 | 3.271 | 3.274 |
| M8[2222][2222] | Class1 | 16.609 | 96.816 | 65.014 | 64.672 |
| M8[2222][2222] | Class2 | 32.613 | 90.856 | 23.128 | 23.351 |
| M8[2222][2222] | Class3 | 226.860 | 95.601 | 8.638 | 8.743 |
| M8[2222][2222] | Class4 | 949.363 | 96.944 | 3.220 | 3.233 |
| M9[3333][3333] | Class1 | 16.529 | 96.805 | 65.065 | 64.703 |
| M9[3333][3333] | Class2 | 32.704 | 90.869 | 23.102 | 23.329 |
| M9[3333][3333] | Class3 | 244.752 | 95.903 | 8.587 | 8.730 |
| M9[3333][3333] | Class4 | 917.973 | 96.847 | 3.246 | 3.237 |

**Supplemental Tables S6.** Trajectory parameter estimation for the model in ELSA.

| **Model ID** | **Traj_Y** | ***Parameter*** | ***Class*** | ***β*** | ***SE*** | ***t*** | ***P*** |
| --- | --- | --- | --- | --- | --- | --- | --- |
| M1[11][11] | Frailty index | Intercept | Class1 | 0.054 | 0.002 | 35.482 | <0.001 |
| M1[11][11] | Frailty index | Linear | Class1 | 0.006 | 0.001 | 11.746 | <0.001 |
| M1[11][11] | Frailty index | Intercept | Class2 | 0.242 | 0.004 | 61.206 | <0.001 |
| M1[11][11] | Frailty index | Linear | Class2 | 0.020 | 0.001 | 15.013 | <0.001 |
| M1[11][11] | CES-D score | Intercept | Class1 | 1.049 | 0.035 | 30.108 | <0.001 |
| M1[11][11] | CES-D score | Linear | Class1 | -0.040 | 0.013 | -3.210 | 0.001 |
| M1[11][11] | CES-D score | Intercept | Class2 | 2.818 | 0.087 | 32.414 | <0.001 |
| M1[11][11] | CES-D score | Linear | Class2 | -0.011 | 0.031 | -0.350 | 0.727 |
| M2[22][22] | Frailty index | Intercept | Class1 | 0.060 | 0.003 | 17.714 | <0.001 |
| M2[22][22] | Frailty index | Linear | Class1 | -0.000 | 0.003 | -0.100 | 0.920 |
| M2[22][22] | Frailty index | Quadratic | Class1 | 0.001 | 0.001 | 2.209 | 0.027 |
| M2[22][22] | Frailty index | Intercept | Class2 | 0.238 | 0.008 | 28.310 | <0.001 |
| M2[22][22] | Frailty index | Linear | Class2 | 0.024 | 0.008 | 3.102 | 0.002 |
| M2[22][22] | Frailty index | Quadratic | Class2 | -0.001 | 0.001 | -0.440 | 0.660 |
| M2[22][22] | CES-D score | Intercept | Class1 | 0.890 | 0.078 | 11.345 | <0.001 |
| M2[22][22] | CES-D score | Linear | Class1 | 0.119 | 0.072 | 1.658 | 0.097 |
| M2[22][22] | CES-D score | Quadratic | Class1 | -0.032 | 0.014 | -2.260 | 0.024 |
| M2[22][22] | CES-D score | Intercept | Class2 | 2.416 | 0.194 | 12.462 | <0.001 |
| M2[22][22] | CES-D score | Linear | Class2 | 0.392 | 0.176 | 2.224 | 0.026 |
| M2[22][22] | CES-D score | Quadratic | Class2 | -0.081 | 0.035 | -2.325 | 0.020 |
| M3[33][33] | Frailty index | Intercept | Class1 | 0.042 | 0.010 | 4.154 | <0.001 |
| M3[33][33] | Frailty index | Linear | Class1 | 0.029 | 0.016 | 1.848 | 0.065 |
| M3[33][33] | Frailty index | Quadratic | Class1 | -0.012 | 0.007 | -1.702 | 0.089 |
| M3[33][33] | Frailty index | Cubic | Class1 | 0.002 | 0.001 | 1.906 | 0.057 |
| M3[33][33] | Frailty index | Intercept | Class2 | 0.150 | 0.025 | 6.085 | <0.001 |
| M3[33][33] | Frailty index | Linear | Class2 | 0.163 | 0.038 | 4.316 | <0.001 |
| M3[33][33] | Frailty index | Quadratic | Class2 | -0.063 | 0.017 | -3.795 | <0.001 |
| M3[33][33] | Frailty index | Cubic | Class2 | 0.008 | 0.002 | 3.772 | <0.001 |
| M3[33][33] | CES-D score | Intercept | Class1 | 0.615 | 0.234 | 2.627 | 0.009 |
| M3[33][33] | CES-D score | Linear | Class1 | 0.555 | 0.358 | 1.552 | 0.121 |
| M3[33][33] | CES-D score | Quadratic | Class1 | -0.228 | 0.158 | -1.441 | 0.149 |
| M3[33][33] | CES-D score | Cubic | Class1 | 0.026 | 0.021 | 1.245 | 0.213 |
| M3[33][33] | CES-D score | Intercept | Class2 | 3.180 | 0.576 | 5.526 | <0.001 |
| M3[33][33] | CES-D score | Linear | Class2 | -0.822 | 0.880 | -0.934 | 0.351 |
| M3[33][33] | CES-D score | Quadratic | Class2 | 0.464 | 0.389 | 1.193 | 0.233 |
| M3[33][33] | CES-D score | Cubic | Class2 | -0.073 | 0.052 | -1.405 | 0.160 |
| M4[111][111] | Frailty index | Intercept | Class1 | 0.044 | 0.001 | 30.918 | <0.001 |
| M4[111][111] | Frailty index | Linear | Class1 | 0.005 | 0.001 | 9.392 | <0.001 |
| M4[111][111] | Frailty index | Intercept | Class2 | 0.128 | 0.003 | 43.847 | <0.001 |
| M4[111][111] | Frailty index | Linear | Class2 | 0.019 | 0.001 | 18.585 | <0.001 |
| M4[111][111] | Frailty index | Intercept | Class3 | 0.333 | 0.006 | 58.668 | <0.001 |
| M4[111][111] | Frailty index | Linear | Class3 | 0.015 | 0.002 | 8.122 | <0.001 |
| M4[111][111] | CES-D score | Intercept | Class1 | 0.892 | 0.038 | 23.714 | <0.001 |
| M4[111][111] | CES-D score | Linear | Class1 | -0.043 | 0.014 | -3.195 | 0.001 |
| M4[111][111] | CES-D score | Intercept | Class2 | 2.130 | 0.076 | 27.958 | <0.001 |
| M4[111][111] | CES-D score | Linear | Class2 | -0.015 | 0.026 | -0.559 | 0.576 |
| M4[111][111] | CES-D score | Intercept | Class3 | 3.182 | 0.123 | 25.796 | <0.001 |
| M4[111][111] | CES-D score | Linear | Class3 | -0.028 | 0.044 | -0.623 | 0.533 |
| M5[222][222] | Frailty index | Intercept | Class1 | 0.049 | 0.003 | 15.703 | <0.001 |
| M5[222][222] | Frailty index | Linear | Class1 | -0.001 | 0.003 | -0.333 | 0.739 |
| M5[222][222] | Frailty index | Quadratic | Class1 | 0.001 | 0.001 | 2.029 | 0.042 |
| M5[222][222] | Frailty index | Intercept | Class2 | 0.135 | 0.006 | 22.223 | <0.001 |
| M5[222][222] | Frailty index | Linear | Class2 | 0.012 | 0.006 | 2.166 | 0.030 |
| M5[222][222] | Frailty index | Quadratic | Class2 | 0.001 | 0.001 | 1.331 | 0.183 |
| M5[222][222] | Frailty index | Intercept | Class3 | 0.327 | 0.011 | 29.258 | <0.001 |
| M5[222][222] | Frailty index | Linear | Class3 | 0.020 | 0.010 | 2.074 | 0.038 |
| M5[222][222] | Frailty index | Quadratic | Class3 | -0.001 | 0.002 | -0.540 | 0.589 |
| M5[222][222] | CES-D score | Intercept | Class1 | 0.760 | 0.084 | 9.053 | <0.001 |
| M5[222][222] | CES-D score | Linear | Class1 | 0.087 | 0.076 | 1.142 | 0.254 |
| M5[222][222] | CES-D score | Quadratic | Class1 | -0.026 | 0.015 | -1.734 | 0.083 |
| M5[222][222] | CES-D score | Intercept | Class2 | 1.798 | 0.162 | 11.128 | <0.001 |
| M5[222][222] | CES-D score | Linear | Class2 | 0.317 | 0.147 | 2.159 | 0.031 |
| M5[222][222] | CES-D score | Quadratic | Class2 | -0.066 | 0.029 | -2.298 | 0.022 |
| M5[222][222] | CES-D score | Intercept | Class3 | 2.730 | 0.275 | 9.909 | <0.001 |
| M5[222][222] | CES-D score | Linear | Class3 | 0.424 | 0.251 | 1.691 | 0.091 |
| M5[222][222] | CES-D score | Quadratic | Class3 | -0.090 | 0.049 | -1.830 | 0.067 |
| M6[333][333] | Frailty index | Intercept | Class1 | 0.040 | 0.009 | 4.284 | <0.001 |
| M6[333][333] | Frailty index | Linear | Class1 | 0.014 | 0.014 | 1.000 | 0.317 |
| M6[333][333] | Frailty index | Quadratic | Class1 | -0.006 | 0.006 | -0.901 | 0.367 |
| M6[333][333] | Frailty index | Cubic | Class1 | 0.001 | 0.001 | 1.086 | 0.277 |
| M6[333][333] | Frailty index | Intercept | Class2 | 0.071 | 0.018 | 4.012 | <0.001 |
| M6[333][333] | Frailty index | Linear | Class2 | 0.113 | 0.027 | 4.152 | <0.001 |
| M6[333][333] | Frailty index | Quadratic | Class2 | -0.044 | 0.012 | -3.650 | <0.001 |
| M6[333][333] | Frailty index | Cubic | Class2 | 0.006 | 0.002 | 3.784 | <0.001 |
| M6[333][333] | Frailty index | Intercept | Class3 | 0.209 | 0.031 | 6.743 | <0.001 |
| M6[333][333] | Frailty index | Linear | Class3 | 0.210 | 0.048 | 4.421 | <0.001 |
| M6[333][333] | Frailty index | Quadratic | Class3 | -0.087 | 0.021 | -4.112 | <0.001 |
| M6[333][333] | Frailty index | Cubic | Class3 | 0.011 | 0.003 | 4.078 | <0.001 |
| M6[333][333] | CES-D score | Intercept | Class1 | 0.400 | 0.250 | 1.600 | 0.110 |
| M6[333][333] | CES-D score | Linear | Class1 | 0.661 | 0.382 | 1.728 | 0.084 |
| M6[333][333] | CES-D score | Quadratic | Class1 | -0.283 | 0.169 | -1.677 | 0.093 |
| M6[333][333] | CES-D score | Cubic | Class1 | 0.034 | 0.022 | 1.528 | 0.127 |
| M6[333][333] | CES-D score | Intercept | Class2 | 2.280 | 0.481 | 4.743 | <0.001 |
| M6[333][333] | CES-D score | Linear | Class2 | -0.448 | 0.733 | -0.611 | 0.541 |
| M6[333][333] | CES-D score | Quadratic | Class2 | 0.277 | 0.324 | 0.856 | 0.392 |
| M6[333][333] | CES-D score | Cubic | Class2 | -0.046 | 0.043 | -1.066 | 0.287 |
| M6[333][333] | CES-D score | Intercept | Class3 | 3.281 | 0.816 | 4.023 | <0.001 |
| M6[333][333] | CES-D score | Linear | Class3 | -0.440 | 1.248 | -0.353 | 0.724 |
| M6[333][333] | CES-D score | Quadratic | Class3 | 0.294 | 0.552 | 0.533 | 0.594 |
| M6[333][333] | CES-D score | Cubic | Class3 | -0.051 | 0.073 | -0.695 | 0.487 |
| M7[1111][1111] | Frailty index | Intercept | Class1 | 0.039 | 0.001 | 28.500 | <0.001 |
| M7[1111][1111] | Frailty index | Linear | Class1 | 0.004 | 0.000 | 8.456 | <0.001 |
| M7[1111][1111] | Frailty index | Intercept | Class2 | 0.105 | 0.003 | 41.152 | <0.001 |
| M7[1111][1111] | Frailty index | Linear | Class2 | 0.014 | 0.001 | 15.894 | <0.001 |
| M7[1111][1111] | Frailty index | Intercept | Class3 | 0.208 | 0.006 | 34.775 | <0.001 |
| M7[1111][1111] | Frailty index | Linear | Class3 | 0.025 | 0.002 | 15.542 | <0.001 |
| M7[1111][1111] | Frailty index | Intercept | Class4 | 0.408 | 0.007 | 59.687 | <0.001 |
| M7[1111][1111] | Frailty index | Linear | Class4 | 0.011 | 0.002 | 4.426 | <0.001 |
| M7[1111][1111] | CES-D score | Intercept | Class1 | 0.843 | 0.040 | 20.956 | <0.001 |
| M7[1111][1111] | CES-D score | Linear | Class1 | -0.045 | 0.014 | -3.153 | 0.002 |
| M7[1111][1111] | CES-D score | Intercept | Class2 | 1.876 | 0.073 | 25.753 | <0.001 |
| M7[1111][1111] | CES-D score | Linear | Class2 | -0.036 | 0.025 | -1.455 | 0.146 |
| M7[1111][1111] | CES-D score | Intercept | Class3 | 2.435 | 0.123 | 19.737 | <0.001 |
| M7[1111][1111] | CES-D score | Linear | Class3 | 0.048 | 0.040 | 1.203 | 0.229 |
| M7[1111][1111] | CES-D score | Intercept | Class4 | 3.458 | 0.186 | 18.600 | <0.001 |
| M7[1111][1111] | CES-D score | Linear | Class4 | -0.078 | 0.065 | -1.205 | 0.228 |
| M8[2222][2222] | Frailty index | Intercept | Class1 | 0.044 | 0.003 | 14.560 | <0.001 |
| M8[2222][2222] | Frailty index | Linear | Class1 | -0.001 | 0.003 | -0.326 | 0.744 |
| M8[2222][2222] | Frailty index | Quadratic | Class1 | 0.001 | 0.001 | 1.860 | 0.063 |
| M8[2222][2222] | Frailty index | Intercept | Class2 | 0.118 | 0.005 | 22.159 | <0.001 |
| M8[2222][2222] | Frailty index | Linear | Class2 | 0.002 | 0.005 | 0.375 | 0.708 |
| M8[2222][2222] | Frailty index | Quadratic | Class2 | 0.002 | 0.001 | 2.645 | 0.008 |
| M8[2222][2222] | Frailty index | Intercept | Class3 | 0.202 | 0.009 | 21.430 | <0.001 |
| M8[2222][2222] | Frailty index | Linear | Class3 | 0.032 | 0.008 | 3.989 | <0.001 |
| M8[2222][2222] | Frailty index | Quadratic | Class3 | -0.001 | 0.002 | -0.939 | 0.348 |
| M8[2222][2222] | Frailty index | Intercept | Class4 | 0.403 | 0.015 | 27.592 | <0.001 |
| M8[2222][2222] | Frailty index | Linear | Class4 | 0.017 | 0.013 | 1.287 | 0.198 |
| M8[2222][2222] | Frailty index | Quadratic | Class4 | -0.001 | 0.003 | -0.454 | 0.650 |
| M8[2222][2222] | CES-D score | Intercept | Class1 | 0.720 | 0.090 | 8.018 | <0.001 |
| M8[2222][2222] | CES-D score | Linear | Class1 | 0.079 | 0.082 | 0.968 | 0.333 |
| M8[2222][2222] | CES-D score | Quadratic | Class1 | -0.025 | 0.016 | -1.550 | 0.121 |
| M8[2222][2222] | CES-D score | Intercept | Class2 | 1.575 | 0.157 | 10.043 | <0.001 |
| M8[2222][2222] | CES-D score | Linear | Class2 | 0.263 | 0.142 | 1.855 | 0.064 |
| M8[2222][2222] | CES-D score | Quadratic | Class2 | -0.060 | 0.028 | -2.141 | 0.032 |
| M8[2222][2222] | CES-D score | Intercept | Class3 | 2.131 | 0.258 | 8.267 | <0.001 |
| M8[2222][2222] | CES-D score | Linear | Class3 | 0.370 | 0.229 | 1.617 | 0.106 |
| M8[2222][2222] | CES-D score | Quadratic | Class3 | -0.065 | 0.045 | -1.438 | 0.150 |
| M8[2222][2222] | CES-D score | Intercept | Class4 | 2.940 | 0.414 | 7.108 | <0.001 |
| M8[2222][2222] | CES-D score | Linear | Class4 | 0.430 | 0.370 | 1.161 | 0.246 |
| M8[2222][2222] | CES-D score | Quadratic | Class4 | -0.101 | 0.073 | -1.392 | 0.164 |
| M9[3333][3333] | Frailty index | Intercept | Class1 | 0.035 | 0.009 | 3.924 | <0.001 |
| M9[3333][3333] | Frailty index | Linear | Class1 | 0.013 | 0.014 | 0.978 | 0.328 |
| M9[3333][3333] | Frailty index | Quadratic | Class1 | -0.005 | 0.006 | -0.893 | 0.372 |
| M9[3333][3333] | Frailty index | Cubic | Class1 | 0.001 | 0.001 | 1.063 | 0.288 |
| M9[3333][3333] | Frailty index | Intercept | Class2 | 0.062 | 0.015 | 4.017 | <0.001 |
| M9[3333][3333] | Frailty index | Linear | Class2 | 0.091 | 0.024 | 3.881 | <0.001 |
| M9[3333][3333] | Frailty index | Quadratic | Class2 | -0.038 | 0.010 | -3.630 | <0.001 |
| M9[3333][3333] | Frailty index | Cubic | Class2 | 0.005 | 0.001 | 3.881 | <0.001 |
| M9[3333][3333] | Frailty index | Intercept | Class3 | 0.155 | 0.025 | 6.097 | <0.001 |
| M9[3333][3333] | Frailty index | Linear | Class3 | 0.106 | 0.039 | 2.726 | 0.006 |
| M9[3333][3333] | Frailty index | Quadratic | Class3 | -0.035 | 0.017 | -2.011 | 0.044 |
| M9[3333][3333] | Frailty index | Cubic | Class3 | 0.004 | 0.002 | 1.925 | 0.054 |
| M9[3333][3333] | Frailty index | Intercept | Class4 | 0.234 | 0.042 | 5.625 | <0.001 |
| M9[3333][3333] | Frailty index | Linear | Class4 | 0.286 | 0.063 | 4.516 | <0.001 |
| M9[3333][3333] | Frailty index | Quadratic | Class4 | -0.122 | 0.028 | -4.365 | <0.001 |
| M9[3333][3333] | Frailty index | Cubic | Class4 | 0.016 | 0.004 | 4.342 | <0.001 |
| M9[3333][3333] | CES-D score | Intercept | Class1 | 0.334 | 0.267 | 1.251 | 0.211 |
| M9[3333][3333] | CES-D score | Linear | Class1 | 0.693 | 0.408 | 1.699 | 0.089 |
| M9[3333][3333] | CES-D score | Quadratic | Class1 | -0.300 | 0.180 | -1.666 | 0.096 |
| M9[3333][3333] | CES-D score | Cubic | Class1 | 0.037 | 0.024 | 1.532 | 0.125 |
| M9[3333][3333] | CES-D score | Intercept | Class2 | 1.870 | 0.464 | 4.028 | <0.001 |
| M9[3333][3333] | CES-D score | Linear | Class2 | -0.205 | 0.707 | -0.290 | 0.772 |
| M9[3333][3333] | CES-D score | Quadratic | Class2 | 0.149 | 0.312 | 0.478 | 0.632 |
| M9[3333][3333] | CES-D score | Cubic | Class2 | -0.028 | 0.041 | -0.670 | 0.503 |
| M9[3333][3333] | CES-D score | Intercept | Class3 | 2.905 | 0.753 | 3.857 | <0.001 |
| M9[3333][3333] | CES-D score | Linear | Class3 | -0.850 | 1.141 | -0.744 | 0.457 |
| M9[3333][3333] | CES-D score | Quadratic | Class3 | 0.481 | 0.504 | 0.955 | 0.339 |
| M9[3333][3333] | CES-D score | Cubic | Class3 | -0.073 | 0.067 | -1.086 | 0.277 |
| M9[3333][3333] | CES-D score | Intercept | Class4 | 2.686 | 1.213 | 2.214 | 0.027 |
| M9[3333][3333] | CES-D score | Linear | Class4 | 0.803 | 1.842 | 0.436 | 0.663 |
| M9[3333][3333] | CES-D score | Quadratic | Class4 | -0.264 | 0.812 | -0.325 | 0.746 |
| M9[3333][3333] | CES-D score | Cubic | Class4 | 0.021 | 0.108 | 0.197 | 0.844 |

**Supplemental Tables S7**. The best trajectory model applicability in ELSA.

| **GROUP** | **shape** | **avepp** | **COUNT** | **Pj** | **πj** | **occ_c** | **entropy_K** |
| --- | --- | --- | --- | --- | --- | --- | --- |
| Group1 | Linear | 97.954 | 2840 | 72.579 | 72.132 | 18.496 | 0.936 |
| Group2 | Linear | 94.038 | 804 | 20.547 | 20.970 | 59.448 |  |
| Group3 | Linear | 97.628 | 269 | 6.875 | 6.898 | 555.503 |  |

**Supplemental Tables S8**. Evaluation Indicators of the fitting effect of multiple models in HRS.

| **Model ID** | ***Log(LL)*** | ***BIC*** | ***AIC*** | ***E_k_*** |
| --- | --- | --- | --- | --- |
| M1[11][11] | -10635.719 | -10681.086 | -10646.719 | 0.950 |
| M2[22][22] | -10626.421 | -10688.285 | -10641.421 | 0.950 |
| M3[33][33] | -10619.644 | -10698.005 | -10638.644 | 0.950 |
| M4[111][111] | -8718.877 | -8784.865 | -8734.877 | 0.925 |
| M5[222][222] | -8705.610 | -8796.344 | -8727.610 | 0.925 |
| M6[333][333] | -8697.553 | -8813.033 | -8725.553 | 0.925 |
| M7[1111][1111] | -7988.006 | -8074.615 | -8009.006 | 0.903 |
| M8[2222][2222] | -7972.176 | -8091.779 | -8001.176 | 0.903 |
| M9[3333][3333] | -7375.404 | -7528.001 | -7412.404 | 0.937 |

**Supplemental Tables S9**. Multiple model trajectory group evaluation indicators in HRS.

| **Model ID** | **Class** | ***OCC*** | ***AvepP*** | ***P*** | ***π*** |
| --- | --- | --- | --- | --- | --- |
| M1[11][11] | Class1 | 23.156 | 99.144 | 83.307 | 83.339 |
| M1[11][11] | Class2 | 107.083 | 95.537 | 16.693 | 16.661 |
| M2[22][22] | Class1 | 23.196 | 99.146 | 83.307 | 83.345 |
| M2[22][22] | Class2 | 106.409 | 95.508 | 16.693 | 16.655 |
| M3[33][33] | Class1 | 22.765 | 99.129 | 83.333 | 83.331 |
| M3[33][33] | Class2 | 110.229 | 95.662 | 16.667 | 16.669 |
| M4[111][111] | Class1 | 18.481 | 97.576 | 68.838 | 68.539 |
| M4[111][111] | Class2 | 44.524 | 93.709 | 24.751 | 25.067 |
| M4[111][111] | Class3 | 443.519 | 96.805 | 6.410 | 6.394 |
| M5[222][222] | Class1 | 18.637 | 97.596 | 68.812 | 68.535 |
| M5[222][222] | Class2 | 45.160 | 93.793 | 24.725 | 25.072 |
| M5[222][222] | Class3 | 402.920 | 96.494 | 6.463 | 6.394 |
| M6[333][333] | Class1 | 18.755 | 97.610 | 68.786 | 68.527 |
| M6[333][333] | Class2 | 44.934 | 93.766 | 24.751 | 25.079 |
| M6[333][333] | Class3 | 403.245 | 96.497 | 6.463 | 6.394 |
| M7[1111][1111] | Class1 | 15.960 | 96.336 | 62.507 | 62.227 |
| M7[1111][1111] | Class2 | 126.363 | 92.232 | 8.713 | 8.589 |
| M7[1111][1111] | Class3 | 26.228 | 89.746 | 24.673 | 25.020 |
| M7[1111][1111] | Class4 | 1313.604 | 98.278 | 4.108 | 4.164 |
| M8[2222][2222] | Class1 | 15.822 | 96.297 | 62.507 | 62.174 |
| M8[2222][2222] | Class2 | 26.551 | 89.882 | 24.647 | 25.070 |
| M8[2222][2222] | Class3 | 123.594 | 92.081 | 8.739 | 8.599 |
| M8[2222][2222] | Class4 | 1256.808 | 98.199 | 4.108 | 4.157 |
| M9[3333][3333] | Class1 | 162.717 | 92.475 | 6.960 | 7.022 |
| M9[3333][3333] | Class2 | 21.039 | 97.847 | 68.524 | 68.351 |
| M9[3333][3333] | Class3 | 51.108 | 92.461 | 19.205 | 19.353 |
| M9[3333][3333] | Class4 | 619.116 | 97.180 | 5.311 | 5.274 |

**Supplemental Tables S10**. Trajectory parameter estimation for the model in HRS.

| **Model ID** | **Traj_Y** | ***Parameter*** | ***Class*** | ***β*** | ***SE*** | ***t*** | ***P*** |
| --- | --- | --- | --- | --- | --- | --- | --- |
| M1[11][11] | Frailty index | Intercept | Class1 | 0.000 | 0.005 | 0.078 | 0.938 |
| M1[11][11] | Frailty index | Linear | Class1 | 0.008 | 0.001 | 13.879 | <0.001 |
| M1[11][11] | Frailty index | Intercept | Class2 | 0.038 | 0.011 | 3.400 | 0.001 |
| M1[11][11] | Frailty index | Linear | Class2 | 0.026 | 0.001 | 19.918 | <0.001 |
| M1[11][11] | CES-D score | Intercept | Class1 | 0.686 | 0.103 | 6.636 | <0.001 |
| M1[11][11] | CES-D score | Linear | Class1 | 0.000 | 0.012 | 0.010 | 0.992 |
| M1[11][11] | CES-D score | Intercept | Class2 | 2.925 | 0.239 | 12.230 | <0.001 |
| M1[11][11] | CES-D score | Linear | Class2 | -0.057 | 0.028 | -2.068 | 0.039 |
| M2[22][22] | Frailty index | Intercept | Class1 | 0.155 | 0.045 | 3.474 | 0.001 |
| M2[22][22] | Frailty index | Linear | Class1 | -0.029 | 0.011 | -2.748 | 0.006 |
| M2[22][22] | Frailty index | Quadratic | Class1 | 0.002 | 0.001 | 3.486 | <0.001 |
| M2[22][22] | Frailty index | Intercept | Class2 | 0.266 | 0.102 | 2.605 | 0.009 |
| M2[22][22] | Frailty index | Linear | Class2 | -0.029 | 0.024 | -1.185 | 0.236 |
| M2[22][22] | Frailty index | Quadratic | Class2 | 0.003 | 0.001 | 2.249 | 0.025 |
| M2[22][22] | CES-D score | Intercept | Class1 | -0.204 | 0.961 | -0.212 | 0.832 |
| M2[22][22] | CES-D score | Linear | Class1 | 0.213 | 0.229 | 0.930 | 0.352 |
| M2[22][22] | CES-D score | Quadratic | Class1 | -0.013 | 0.013 | -0.931 | 0.352 |
| M2[22][22] | CES-D score | Intercept | Class2 | 3.893 | 2.213 | 1.759 | 0.079 |
| M2[22][22] | CES-D score | Linear | Class2 | -0.289 | 0.527 | -0.548 | 0.584 |
| M2[22][22] | CES-D score | Quadratic | Class2 | 0.014 | 0.031 | 0.439 | 0.660 |
| M3[33][33] | Frailty index | Intercept | Class1 | -0.442 | 0.557 | -0.794 | 0.427 |
| M3[33][33] | Frailty index | Linear | Class1 | 0.186 | 0.200 | 0.928 | 0.353 |
| M3[33][33] | Frailty index | Quadratic | Class1 | -0.023 | 0.024 | -0.983 | 0.325 |
| M3[33][33] | Frailty index | Cubic | Class1 | 0.001 | 0.001 | 1.075 | 0.282 |
| M3[33][33] | Frailty index | Intercept | Class2 | -3.046 | 1.275 | -2.389 | 0.017 |
| M3[33][33] | Frailty index | Linear | Class2 | 1.163 | 0.458 | 2.539 | 0.011 |
| M3[33][33] | Frailty index | Quadratic | Class2 | -0.138 | 0.054 | -2.546 | 0.011 |
| M3[33][33] | Frailty index | Cubic | Class2 | 0.006 | 0.002 | 2.607 | 0.009 |
| M3[33][33] | CES-D score | Intercept | Class1 | -1.790 | 12.012 | -0.149 | 0.882 |
| M3[33][33] | CES-D score | Linear | Class1 | 0.784 | 4.314 | 0.182 | 0.856 |
| M3[33][33] | CES-D score | Quadratic | Class1 | -0.080 | 0.512 | -0.157 | 0.875 |
| M3[33][33] | CES-D score | Cubic | Class1 | 0.003 | 0.020 | 0.133 | 0.894 |
| M3[33][33] | CES-D score | Intercept | Class2 | -60.456 | 27.853 | -2.171 | 0.030 |
| M3[33][33] | CES-D score | Linear | Class2 | 22.862 | 10.002 | 2.286 | 0.022 |
| M3[33][33] | CES-D score | Quadratic | Class2 | -2.736 | 1.186 | -2.306 | 0.021 |
| M3[33][33] | CES-D score | Cubic | Class2 | 0.108 | 0.046 | 2.319 | 0.020 |
| M4[111][111] | Frailty index | Intercept | Class1 | 0.003 | 0.004 | 0.644 | 0.520 |
| M4[111][111] | Frailty index | Linear | Class1 | 0.006 | 0.001 | 10.789 | <0.001 |
| M4[111][111] | Frailty index | Intercept | Class2 | 0.011 | 0.008 | 1.396 | 0.163 |
| M4[111][111] | Frailty index | Linear | Class2 | 0.018 | 0.001 | 20.536 | <0.001 |
| M4[111][111] | Frailty index | Intercept | Class3 | 0.030 | 0.015 | 2.010 | 0.044 |
| M4[111][111] | Frailty index | Linear | Class3 | 0.037 | 0.002 | 20.607 | <0.001 |
| M4[111][111] | CES-D score | Intercept | Class1 | 0.670 | 0.114 | 5.891 | <0.001 |
| M4[111][111] | CES-D score | Linear | Class1 | -0.009 | 0.013 | -0.702 | 0.483 |
| M4[111][111] | CES-D score | Intercept | Class2 | 1.535 | 0.195 | 7.864 | <0.001 |
| M4[111][111] | CES-D score | Linear | Class2 | -0.005 | 0.023 | -0.220 | 0.826 |
| M4[111][111] | CES-D score | Intercept | Class3 | 3.354 | 0.380 | 8.818 | <0.001 |
| M4[111][111] | CES-D score | Linear | Class3 | -0.028 | 0.044 | -0.645 | 0.519 |
| M5[222][222] | Frailty index | Intercept | Class1 | 0.167 | 0.041 | 4.078 | <0.001 |
| M5[222][222] | Frailty index | Linear | Class1 | -0.034 | 0.010 | -3.451 | 0.001 |
| M5[222][222] | Frailty index | Quadratic | Class1 | 0.002 | 0.001 | 4.030 | <0.001 |
| M5[222][222] | Frailty index | Intercept | Class2 | 0.155 | 0.070 | 2.230 | 0.026 |
| M5[222][222] | Frailty index | Linear | Class2 | -0.016 | 0.017 | -0.976 | 0.329 |
| M5[222][222] | Frailty index | Quadratic | Class2 | 0.002 | 0.001 | 2.086 | 0.037 |
| M5[222][222] | Frailty index | Intercept | Class3 | 0.313 | 0.138 | 2.276 | 0.023 |
| M5[222][222] | Frailty index | Linear | Class3 | -0.031 | 0.033 | -0.949 | 0.343 |
| M5[222][222] | Frailty index | Quadratic | Class3 | 0.004 | 0.002 | 2.068 | 0.039 |
| M5[222][222] | CES-D score | Intercept | Class1 | -0.204 | 1.057 | -0.193 | 0.847 |
| M5[222][222] | CES-D score | Linear | Class1 | 0.200 | 0.252 | 0.794 | 0.427 |
| M5[222][222] | CES-D score | Quadratic | Class1 | -0.012 | 0.015 | -0.832 | 0.405 |
| M5[222][222] | CES-D score | Intercept | Class2 | 1.504 | 1.793 | 0.838 | 0.402 |
| M5[222][222] | CES-D score | Linear | Class2 | 0.002 | 0.427 | 0.006 | 0.995 |
| M5[222][222] | CES-D score | Quadratic | Class2 | -0.000 | 0.025 | -0.017 | 0.986 |
| M5[222][222] | CES-D score | Intercept | Class3 | 3.767 | 3.497 | 1.077 | 0.281 |
| M5[222][222] | CES-D score | Linear | Class3 | -0.128 | 0.833 | -0.153 | 0.878 |
| M5[222][222] | CES-D score | Quadratic | Class3 | 0.006 | 0.049 | 0.119 | 0.905 |
| M6[333][333] | Frailty index | Intercept | Class1 | -0.089 | 0.511 | -0.174 | 0.862 |
| M6[333][333] | Frailty index | Linear | Class1 | 0.058 | 0.184 | 0.318 | 0.751 |
| M6[333][333] | Frailty index | Quadratic | Class1 | -0.009 | 0.022 | -0.395 | 0.693 |
| M6[333][333] | Frailty index | Cubic | Class1 | 0.000 | 0.001 | 0.501 | 0.616 |
| M6[333][333] | Frailty index | Intercept | Class2 | -2.730 | 0.864 | -3.160 | 0.002 |
| M6[333][333] | Frailty index | Linear | Class2 | 1.022 | 0.310 | 3.292 | 0.001 |
| M6[333][333] | Frailty index | Quadratic | Class2 | -0.121 | 0.037 | -3.292 | 0.001 |
| M6[333][333] | Frailty index | Cubic | Class2 | 0.005 | 0.001 | 3.348 | 0.001 |
| M6[333][333] | Frailty index | Intercept | Class3 | -2.045 | 1.703 | -1.201 | 0.230 |
| M6[333][333] | Frailty index | Linear | Class3 | 0.817 | 0.612 | 1.336 | 0.181 |
| M6[333][333] | Frailty index | Quadratic | Class3 | -0.097 | 0.073 | -1.333 | 0.182 |
| M6[333][333] | Frailty index | Cubic | Class3 | 0.004 | 0.003 | 1.388 | 0.165 |
| M6[333][333] | CES-D score | Intercept | Class1 | -4.711 | 13.229 | -0.356 | 0.722 |
| M6[333][333] | CES-D score | Linear | Class1 | 1.822 | 4.751 | 0.384 | 0.701 |
| M6[333][333] | CES-D score | Quadratic | Class1 | -0.205 | 0.564 | -0.364 | 0.716 |
| M6[333][333] | CES-D score | Cubic | Class1 | 0.008 | 0.022 | 0.342 | 0.732 |
| M6[333][333] | CES-D score | Intercept | Class2 | -23.573 | 22.490 | -1.048 | 0.295 |
| M6[333][333] | CES-D score | Linear | Class2 | 9.025 | 8.077 | 1.117 | 0.264 |
| M6[333][333] | CES-D score | Quadratic | Class2 | -1.072 | 0.958 | -1.119 | 0.263 |
| M6[333][333] | CES-D score | Cubic | Class2 | 0.042 | 0.038 | 1.119 | 0.263 |
| M6[333][333] | CES-D score | Intercept | Class3 | -37.989 | 43.317 | -0.877 | 0.381 |
| M6[333][333] | CES-D score | Linear | Class3 | 14.897 | 15.562 | 0.957 | 0.338 |
| M6[333][333] | CES-D score | Quadratic | Class3 | -1.779 | 1.847 | -0.963 | 0.336 |
| M6[333][333] | CES-D score | Cubic | Class3 | 0.070 | 0.072 | 0.966 | 0.334 |
| M7[1111][1111] | Frailty index | Intercept | Class1 | 0.004 | 0.004 | 0.809 | 0.419 |
| M7[1111][1111] | Frailty index | Linear | Class1 | 0.005 | 0.001 | 9.491 | <0.001 |
| M7[1111][1111] | Frailty index | Intercept | Class2 | 0.031 | 0.014 | 2.157 | 0.031 |
| M7[1111][1111] | Frailty index | Linear | Class2 | 0.025 | 0.002 | 14.946 | <0.001 |
| M7[1111][1111] | Frailty index | Intercept | Class3 | -0.000 | 0.008 | -0.030 | 0.976 |
| M7[1111][1111] | Frailty index | Linear | Class3 | 0.016 | 0.001 | 17.721 | <0.001 |
| M7[1111][1111] | Frailty index | Intercept | Class4 | 0.043 | 0.018 | 2.336 | 0.019 |
| M7[1111][1111] | Frailty index | Linear | Class4 | 0.038 | 0.002 | 17.493 | <0.001 |
| M7[1111][1111] | CES-D score | Intercept | Class1 | 0.626 | 0.117 | 5.346 | <0.001 |
| M7[1111][1111] | CES-D score | Linear | Class1 | -0.014 | 0.014 | -0.993 | 0.321 |
| M7[1111][1111] | CES-D score | Intercept | Class2 | 1.165 | 0.347 | 3.355 | 0.001 |
| M7[1111][1111] | CES-D score | Linear | Class2 | 0.003 | 0.039 | 0.081 | 0.936 |
| M7[1111][1111] | CES-D score | Intercept | Class3 | 1.592 | 0.201 | 7.935 | <0.001 |
| M7[1111][1111] | CES-D score | Linear | Class3 | -0.001 | 0.023 | -0.052 | 0.958 |
| M7[1111][1111] | CES-D score | Intercept | Class4 | 4.107 | 0.467 | 8.797 | <0.001 |
| M7[1111][1111] | CES-D score | Linear | Class4 | -0.024 | 0.054 | -0.443 | 0.658 |
| M8[2222][2222] | Frailty index | Intercept | Class1 | 0.164 | 0.041 | 4.019 | <0.001 |
| M8[2222][2222] | Frailty index | Linear | Class1 | -0.033 | 0.010 | -3.440 | 0.001 |
| M8[2222][2222] | Frailty index | Quadratic | Class1 | 0.002 | 0.001 | 3.952 | <0.001 |
| M8[2222][2222] | Frailty index | Intercept | Class2 | 0.131 | 0.068 | 1.936 | 0.053 |
| M8[2222][2222] | Frailty index | Linear | Class2 | -0.016 | 0.016 | -0.967 | 0.333 |
| M8[2222][2222] | Frailty index | Quadratic | Class2 | 0.002 | 0.001 | 1.953 | 0.051 |
| M8[2222][2222] | Frailty index | Intercept | Class3 | 0.297 | 0.120 | 2.481 | 0.013 |
| M8[2222][2222] | Frailty index | Linear | Class3 | -0.038 | 0.028 | -1.350 | 0.177 |
| M8[2222][2222] | Frailty index | Quadratic | Class3 | 0.004 | 0.002 | 2.239 | 0.025 |
| M8[2222][2222] | Frailty index | Intercept | Class4 | 0.316 | 0.163 | 1.934 | 0.053 |
| M8[2222][2222] | Frailty index | Linear | Class4 | -0.027 | 0.039 | -0.702 | 0.483 |
| M8[2222][2222] | Frailty index | Quadratic | Class4 | 0.004 | 0.002 | 1.682 | 0.093 |
| M8[2222][2222] | CES-D score | Intercept | Class1 | -0.238 | 1.086 | -0.220 | 0.826 |
| M8[2222][2222] | CES-D score | Linear | Class1 | 0.193 | 0.259 | 0.747 | 0.455 |
| M8[2222][2222] | CES-D score | Quadratic | Class1 | -0.012 | 0.015 | -0.801 | 0.423 |
| M8[2222][2222] | CES-D score | Intercept | Class2 | 0.415 | 1.780 | 0.233 | 0.816 |
| M8[2222][2222] | CES-D score | Linear | Class2 | 0.279 | 0.424 | 0.659 | 0.510 |
| M8[2222][2222] | CES-D score | Quadratic | Class2 | -0.016 | 0.025 | -0.661 | 0.508 |
| M8[2222][2222] | CES-D score | Intercept | Class3 | 2.096 | 2.996 | 0.700 | 0.484 |
| M8[2222][2222] | CES-D score | Linear | Class3 | -0.217 | 0.713 | -0.304 | 0.761 |
| M8[2222][2222] | CES-D score | Quadratic | Class3 | 0.013 | 0.042 | 0.307 | 0.759 |
| M8[2222][2222] | CES-D score | Intercept | Class4 | 8.225 | 4.210 | 1.954 | 0.051 |
| M8[2222][2222] | CES-D score | Linear | Class4 | -1.012 | 1.003 | -1.009 | 0.313 |
| M8[2222][2222] | CES-D score | Quadratic | Class4 | 0.058 | 0.059 | 0.987 | 0.323 |
| M9[3333][3333] | Frailty index | Intercept | Class1 | -1.924 | 1.678 | -1.147 | 0.252 |
| M9[3333][3333] | Frailty index | Linear | Class1 | 0.725 | 0.603 | 1.202 | 0.229 |
| M9[3333][3333] | Frailty index | Quadratic | Class1 | -0.086 | 0.072 | -1.202 | 0.229 |
| M9[3333][3333] | Frailty index | Cubic | Class1 | 0.003 | 0.003 | 1.218 | 0.223 |
| M9[3333][3333] | Frailty index | Intercept | Class2 | -0.095 | 0.522 | -0.183 | 0.855 |
| M9[3333][3333] | Frailty index | Linear | Class2 | 0.060 | 0.187 | 0.321 | 0.748 |
| M9[3333][3333] | Frailty index | Quadratic | Class2 | -0.009 | 0.022 | -0.394 | 0.694 |
| M9[3333][3333] | Frailty index | Cubic | Class2 | 0.000 | 0.001 | 0.496 | 0.620 |
| M9[3333][3333] | Frailty index | Intercept | Class3 | -2.983 | 1.023 | -2.916 | 0.004 |
| M9[3333][3333] | Frailty index | Linear | Class3 | 1.122 | 0.367 | 3.052 | 0.002 |
| M9[3333][3333] | Frailty index | Quadratic | Class3 | -0.134 | 0.044 | -3.066 | 0.002 |
| M9[3333][3333] | Frailty index | Cubic | Class3 | 0.005 | 0.002 | 3.131 | 0.002 |
| M9[3333][3333] | Frailty index | Intercept | Class4 | -1.874 | 1.917 | -0.978 | 0.328 |
| M9[3333][3333] | Frailty index | Linear | Class4 | 0.749 | 0.688 | 1.088 | 0.277 |
| M9[3333][3333] | Frailty index | Quadratic | Class4 | -0.088 | 0.082 | -1.074 | 0.283 |
| M9[3333][3333] | Frailty index | Cubic | Class4 | 0.004 | 0.003 | 1.116 | 0.265 |
| M9[3333][3333] | CES-D score | Intercept | Class1 | -124.242 | 42.993 | -2.890 | 0.004 |
| M9[3333][3333] | CES-D score | Linear | Class1 | 45.737 | 15.444 | 2.962 | 0.003 |
| M9[3333][3333] | CES-D score | Quadratic | Class1 | -5.379 | 1.833 | -2.935 | 0.003 |
| M9[3333][3333] | CES-D score | Cubic | Class1 | 0.208 | 0.072 | 2.900 | 0.004 |
| M9[3333][3333] | CES-D score | Intercept | Class2 | 0.247 | 11.550 | 0.021 | 0.983 |
| M9[3333][3333] | CES-D score | Linear | Class2 | 0.030 | 4.148 | 0.007 | 0.994 |
| M9[3333][3333] | CES-D score | Quadratic | Class2 | 0.006 | 0.492 | 0.012 | 0.990 |
| M9[3333][3333] | CES-D score | Cubic | Class2 | -0.001 | 0.019 | -0.033 | 0.974 |
| M9[3333][3333] | CES-D score | Intercept | Class3 | -8.276 | 22.894 | -0.361 | 0.718 |
| M9[3333][3333] | CES-D score | Linear | Class3 | 3.386 | 8.223 | 0.412 | 0.681 |
| M9[3333][3333] | CES-D score | Quadratic | Class3 | -0.415 | 0.976 | -0.425 | 0.671 |
| M9[3333][3333] | CES-D score | Cubic | Class3 | 0.017 | 0.038 | 0.440 | 0.660 |
| M9[3333][3333] | CES-D score | Intercept | Class4 | -26.776 | 42.584 | -0.629 | 0.530 |
| M9[3333][3333] | CES-D score | Linear | Class4 | 10.947 | 15.294 | 0.716 | 0.474 |
| M9[3333][3333] | CES-D score | Quadratic | Class4 | -1.311 | 1.815 | -0.723 | 0.470 |
| M9[3333][3333] | CES-D score | Cubic | Class4 | 0.052 | 0.071 | 0.728 | 0.466 |

**Supplemental Tables S11**. Best trajectory model applicability in HRS.

| **GROUP** | **shape** | **avepp** | **COUNT** | **Pj** | **πj** | **occ_c** | **entropy_K** |
| --- | --- | --- | --- | --- | --- | --- | --- |
| Group1 | Linear | 97.576 | 2631 | 68.838 | 68.539 | 18.481 | 0.925 |
| Group2 | Linear | 93.709 | 946 | 24.751 | 25.067 | 44.524 |  |
| Group3 | Linear | 96.805 | 245 | 6.410 | 6.394 | 443.519 |  |

**Supplemental Tables S12**. Sensitivity analysis of the association between joint trajectories of frailty index and depressive symptoms and incident OA using multivariable logistic regression.

| Variables | Model1 | |  | Model2 | |  | Model3 | |
| --- | --- | --- | --- | --- | --- | --- | --- | --- |
|  | OR (95%CI) | *P* |  | OR (95%CI) | *P* |  | OR (95%CI) | *P* |
| ELSA | | | | | | |  |  |
| GROUP |  |  |  |  |  |  |  |  |
| 1 | 1.00 (Reference) |  |  | 1.00 (Reference) |  |  | 1.00 (Reference) |  |
| 2 | 2.13 (1.80 ~ 2.52) | **<.001** |  | 2.00 (1.68 ~ 2.39) | **<.001** |  | 1.94 (1.61 ~ 2.34) | **<.001** |
| 3 | 4.03 (3.12 ~ 5.21) | **<.001** |  | 3.78 (2.90 ~ 4.92) | **<.001** |  | 3.73 (2.78 ~ 5.00) | **<.001** |
| HRS | | | | | | |  |  |
| GROUP |  |  |  |  |  |  |  |  |
| 1 | 1.00 (Reference) |  |  | 1.00 (Reference) |  |  | 1.00 (Reference) |  |
| 2 | 1.20 (1.01 ~ 1.42) | **0.043** |  | 1.30 (1.09 ~ 1.56) | **0.003** |  | 1.38 (1.14 ~ 1.66) | **<.001** |
| 3 | 1.36 (1.01 ~ 1.82) | **0.041** |  | 1.42 (1.05 ~ 1.91) | **0.021** |  | 1.67 (1.22 ~ 2.29) | **0.001** |
| OR: Odds Ratio, CI: Confidence Interval | | | | | | |  |  |
| Model1: Crude | | | | | | |  |  |
| Model2: Adjust: Age, Gender | | | | | | |  |  |
| Model3: Adjust: Age, Gender, Race, Marital, Education, Smoke, Drink, BMI, Physical activity | | | | | | |  |  |

**Supplemental Tables S13.** Sensitivity analysis of the association between joint trajectories of frailty index and depressive symptoms and incident OA after excluding participants who developed OA during the first follow-up wave.

| Variables | Model1 | |  | Model2 | |  | Model3 | |  |  |
| --- | --- | --- | --- | --- | --- | --- | --- | --- | --- | --- |
|  | HR (95%CI) | *P* | HR (95%CI) |  |  | *P* |  |  | HR (95%CI) | *P* |
| ELSA | | | | | | |  |  |  |  |
| GROUP |  |  |  |  |  |  |  |  |  |  |
| 1 | 1.00 (Reference) |  |  | 1.00 (Reference) |  |  | 1.00 (Reference) |  |  |  |
| 2 | 1.97 (1.64 ~ 2.37) | **<.001** |  | 1.75 (1.45 ~ 2.12) | **<.001** |  | 1.66 (1.36 ~ 2.02) | **<.001** |  |  |
| 3 | 3.80 (2.93 ~ 4.93) | **<.001** |  | 3.38 (2.59 ~ 4.40) | **<.001** |  | 3.17 (2.37 ~ 4.25) | **<.001** |  |  |
| HRS | | | | | | |  |  |  |  |
| GROUP |  |  |  |  |  |  |  |  |  |  |
| 1 | 1.00 (Reference) |  |  | 1.00 (Reference) |  |  | 1.00 (Reference) |  |  |  |
| 2 | 1.41 (1.18 ~ 1.69) | **<.001** |  | 1.37 (1.14 ~ 1.64) | **<.001** |  | 1.37 (1.13 ~ 1.66) | **<.001** |  |  |
| 3 | 1.98 (1.45 ~ 2.69) | **<.001** |  | 1.95 (1.43 ~ 2.66) | **<.001** |  | 2.02 (1.46 ~ 2.81) | **<.001** |  |  |
| HR: Hazard Ratio, CI: Confidence Interval | | | | | | |  |  |  |  |
| Model1: Crude | | | | | | |  |  |  |  |
| Model2: Adjust: Age, Gender | | | | | | |  |  |  |  |
| Model3: Adjust: Age, Gender, Race, Marital, Education, Smoke, Drink, BMI, Physical activity | | | | | | |  |  |  |  |

**Supplemental Tables S14.** Sensitivity analysis of the association between joint trajectories of frailty index and depressive symptoms and incident OA using complete-case analysis without imputation of missing covariate data.

| Variables | Model1 | |  | Model2 | |  | Model3 | |  |  |
| --- | --- | --- | --- | --- | --- | --- | --- | --- | --- | --- |
|  | HR (95%CI) | *P* | HR (95%CI) |  |  | *P* |  |  | HR (95%CI) | *P* |
| ELSA | | | | | | |  |  |  |  |
| GROUP |  |  |  |  |  |  |  |  |  |  |
| 1 | 1.00 (Reference) |  |  | 1.00 (Reference) |  |  | 1.00 (Reference) |  |  |  |
| 2 | 2.26 (1.96 ~ 2.60) | **<.001** |  | 2.00 (1.73 ~ 2.32) | **<.001** |  | 1.78 (1.48 ~ 2.14) | **<.001** |  |  |
| 3 | 4.30 (3.57 ~ 5.17) | **<.001** |  | 3.74 (3.09 ~ 4.53) | **<.001** |  | 3.30 (2.51 ~ 4.32) | **<.001** |  |  |
| HRS | | | | | | |  |  |  |  |
| GROUP |  |  |  |  |  |  |  |  |  |  |
| 1 | 1.00 (Reference) |  |  | 1.00 (Reference) |  |  | 1.00 (Reference) |  |  |  |
| 2 | 1.48 (1.27 ~ 1.72) | **<.001** |  | 1.45 (1.25 ~ 1.69) | **<.001** |  | 1.52 (1.29 ~ 1.79) | **<.001** |  |  |
| 3 | 2.04 (1.59 ~ 2.63) | **<.001** |  | 2.01 (1.56 ~ 2.58) | **<.001** |  | 2.28 (1.73 ~ 2.99) | **<.001** |  |  |
| HR: Hazard Ratio, CI: Confidence Interval | | | | | | |  |  |  |  |
| Model1: Crude | | | | | | |  |  |  |  |
| Model2: Adjust: Age, Gender | | | | | | |  |  |  |  |
| Model3: Adjust:Age, Gender, Race, Marital, Education, Smoke, Drink, BMI, Physical activity | | | | | | |  |  |  |  |

**Supplemental Tables S15.** Sensitivity analysis of the association between joint trajectories of frailty index and depressive symptoms and incident OA using a four-group trajectory model.

| Variables | Model1 | |  | Model2 | |  | Model3 | |  |  |
| --- | --- | --- | --- | --- | --- | --- | --- | --- | --- | --- |
|  | HR (95%CI) | *P* | HR (95%CI) |  |  | *P* |  |  | HR (95%CI) | *P* |
| ELSA | | | | | | |  |  |  |  |
| GROUP |  |  |  |  |  |  |  |  |  |  |
| 1 | 1.00 (Reference) |  |  | 1.00 (Reference) |  |  | 1.00 (Reference) |  |  |  |
| 2 | 2.11 (1.83 ~ 2.44) | **<.001** |  | 1.92 (1.66 ~ 2.23) | **<.001** |  | 1.82 (1.56 ~ 2.12) | **<.001** |  |  |
| 3 | 3.58 (2.99 ~ 4.29) | **<.001** |  | 3.11 (2.58 ~ 3.75) | **<.001** |  | 2.89 (2.35 ~ 3.54) | **<.001** |  |  |
| 4 | 5.51 (4.28 ~ 7.09) | **<.001** |  | 4.80 (3.72 ~ 6.20) | **<.001** |  | 4.57 (3.45 ~ 6.06) | **<.001** |  |  |
| HRS | | | | | | |  |  |  |  |
| GROUP |  |  |  |  |  |  |  |  |  |  |
| 1 | 1.00 (Reference) |  |  | 1.00 (Reference) |  |  | 1.00 (Reference) |  |  |  |
| 2 | 1.72 (1.36 ~ 2.18) | **<.001** |  | 1.69 (1.33 ~ 2.14) | **<.001** |  | 1.80 (1.40 ~ 2.31) | **<.001** |  |  |
| 3 | 1.35 (1.16 ~ 1.57) | **<.001** |  | 1.32 (1.13 ~ 1.54) | **<.001** |  | 1.35 (1.15 ~ 1.59) | **<.001** |  |  |
| 4 | 2.20 (1.63 ~ 2.96) | **<.001** |  | 2.18 (1.61 ~ 2.94) | **<.001** |  | 2.43 (1.77 ~ 3.34) | **<.001** |  |  |
| HR: Hazard Ratio, CI: Confidence Interval | | | | | | |  |  |  |  |
| Model1: Crude | | | | | | |  |  |  |  |
| Model2: Adjust: Age, Gender | | | | | | |  |  |  |  |
| Model3: Adjust: Age, Gender, Race, Marital, Education, Smoke, Drink, BMI, Physical activity | | | | | | |  |  |  |  |

**Supplemental Figure 1.** Sensitivity analysis using a four-group multi-trajectory model of frailty index and depressive symptoms.
